# Supplementary material for: Inhibitory Control of Prefrontal Cortex by the Claustrum
Source: Neuron. 2018 Sep 5;99(5):1029–1039.e4. doi: 10.1016/j.neuron.2018.07.031 (PMC6168643; doi:10.1016/j.neuron.2018.07.031)
Supplement: Document S1. Figures S1–S4 [file mmc1.pdf]

Neuron, Volume *99*

Supplemental Information

Inhibitory Control of Prefrontal Cortex by the Claustrum

Jesse Jackson, Mahesh M. Karnani, Boris V. Zemelman, Denis Burdakov, and Albert K. Lee

Figure S1: Claustrum axons throughout the brain and PFC pyramidal cell and interneuron responses to CLA activation (related to Figure 1).

A: Brain atlas images taken from The Mouse Brain in Stereotaxic Coordinates, 3rd ed., Franklin and Paxinos (Academic Press, 2007). Black boxes indicate the regions that are shown below, with CLA axons containing ChR2, and stained for GFP. ACC = anterior cingulate; M2 =secondary motor cortex; RSC = retrosplenial cortex; BLA = basolateral amygdala; S1 = primary somatosensory cortex; A1 = primary auditory cortex; V1 = primary visual cortex; POS = postsubiculum; PRC = perirhinal cortex; LEC = lateral entorhinal cortex.

B: The distribution of PFC projecting CLA collaterals in 18 different brain regions, averaged across all layers. Control brain regions (striatum, hippocampus and piriform cortex) are shown for reference, as these regions do not receive CLA inputs. Axon intensity in all regions are normalized to the mean GFP intensity in the control regions.

C: Single trial traces of three different interneurons are shown aligned to optogenetic activation of the CLA (blue) in awake mice. These cells are excited by stimulation, and then subsequently suppressed. Note these activated cells begin firing as early as 6ms following CLA activation, and in some cases, cells can fire doublets or triplets of spikes at ~200 Hz.

D: Late activated PCs had a significantly lower baseline firing rate (2.9±0.6 Hz) than late suppressed PCs (5.2±0.9 Hz, $z = 2.3$, $p = 0.02$).

E: There was no significant correlation between the baseline firing rate and the CLA modulation index for PCs.

F: The baseline firing rate was not significantly different between interneurons that were excited and interneurons that were inhibited by CLA activation

G: There was no significant correlation between the baseline firing rate and the CLA modulation index for INs.

H: The distance from the cortical surface was not significantly different between late activated and late suppressed PCs.

I: There was no significant correlation between the PC recording depth and the CLA modulation index.

J: A trend was observed in which the interneurons excited by CLA activation were located in deeper cortical layers relative to inhibited interneurons.

K: A significant correlation between CLA modulation and recording depth was detected for interneurons.

Figure S2: Optogenetic activation of the BLA and MD (related to Figure 1)

A: Schematic shows the injection of AAVretro-syn-Cre into the PFC + AAV5-EF1a-DIO-ChR2-eYFP into the BLA. On the right, the histological coronal sections show the PFC and the injection site in the BLA.

B: The mean PSTH showing the response of PFC PC cells to optogenetic stimulation of the BLA.

C: Schematic showing the injection of AAV1-syn-ChR2 into the dorsomedial thalamus (MD) of a rat. On the right, the histological sections show the PFC and the injection site in the MD.

D: The mean PSTH showing the response of PFC PC cells to optogenetic stimulation of the MD. Data are pooled across recordings from rats and mice.

E: The mean PSTH (all PCs and INs) for control experiments in mice in which AAVretro-syn-Cre was injected to the PFC and AAV5-DIO-eYFP was injected into the CLA.

Laser pulse timing and duration are indicated by the blue line in each panel. The modulation index values of the individual recordings that constitute the average responses in B, C, and E are shown as points in Figure 1L.

Figure S3: Examples of NPY-NGF neurons and NPY cells that exhibited bursting (related to Figure 2 and Figure 3)

A: Example NPY cells showing the response to hyperpolarization (black), just past spike threshold depolarization (green), and >2x spike threshold depolarization (gray). Examples shown in i – iii were not considered NPY-NGF cells as they had rapidly adapting spike trains, fired spikes early during 'just past threshold depolarizations', a hyperpolarization sag, and often showed rebound spiking following hyperpolarization. Cells in iv – viii were considered NPY-NGF cells as they had regular spiking properties and often showed later spiking in response to 'just past threshold depolarization'.

B: The separation between cells classified as putative NPY-NGF and NPY-burster is shown. Cells with an adaptation index of > -0.2 were considered as candidates for NPY-NGF cells. The adaptation index was defined using the strongest depolarization current step and computed as $(FR_{late} – FR_{early}) / (FR_{late}+ FR_{early})$. The more negative values have greater firing rate adaptation. FR_{early} is the firing rate in the first 500ms and FR_{late} is the firing rate in the last 500ms of the current injection.

C-D: The comparison of EPSC magnitude (C) EPSP magnitude (D) in response to CLA optogenetic activation for each NPY cell type.

E: The latency of each NPY cell type to optical CLA activation as measured in voltage-clamp. The NPY-NGF cells are active earlier.

F: The probability that each cell type will exhibit action potentials in response to CLA activation. Only 1 / 9 NPY-burster cells spiked whereas 5/17 NPY-NGF cells showed spikes. Of the NPY-NGF cells which showed definitive 'late spiking', 4/11 showed spiking in response to CLA activation.

G: The EPSP amplitude for pyramidal cells recorded simultaneously with different interneuron sub-types. No significant difference was found, suggesting the different excitatory responses of interneuron sub-types was not due to overall differences in excitability between experiments.

H: The EPSP amplitude as a function of distance from Pia. PV cells (magenta), NPY cells (green), and all neurons recorded (black) are shown. The Pia measurement was the shortest path to the pia mater from the recording electrode in vitro, whereas *in vivo* (Figure 1, Figure S1) the Pia distance was from the brain surface.

Figure S4: Mean responses of PFC pyramidal cells and FS interneurons to CLA activation before and after pharmacogenetic suppression of different interneuron subtypes (related to Figure 4).

A-E: The mean PC spiking PSTHs during CLA activation pre and post suppression of different interneuron subtypes. Data are presented as the median ± interquartile range. The expanded view in C for the PV-hM4D case shows the weak and brief increased excitation following PV cell suppression. NPY inhibition likely arrives shortly after to rapidly suppress any further excitation. Only the median response is plotted in this panel for clarity.

F: PSTH of FS interneurons *in vivo* during optogenetic CLA activation. Note that only a brief increase in spiking is observed.

G: The PSTH of pyramidal cells during optogenetic CLA activation (as shown in Figure 4E).

H: The PSTH of pyramidal cells during brief (1-5ms) optogenetic PV activation with ChR2.

I: The overlaid PSTH data from G and H. Note that the suppression lasts much shorter with PV activation than with CLA activation.

J: PSTH of FS interneurons *in vivo* during optogenetic CLA activation, following the suppression of NPY interneurons using AAV5-FLEX-hM4d + CNO. Note that PV firing is increased for a much longer duration than in F, where NPY neurons are intact.

K: The PSTH of pyramidal cells during optogenetic CLA activation, following the suppression of NPY interneurons using AAV5-FLEX-hM4d + CNO (as shown in Figure 4E).

L: The PSTH of pyramidal cells during long lasting (80ms) optogenetic PV activation with ChR2. The pulse train used was a 50 Hz train for 80ms. This long lasting PV activation is meant to approximate the extended duration of increased PV activity in J, following the suppression of NPY interneurons.

M: The overlayed PSTH data from K and L. Note the extended duration of inhibition with long lasting PV activation that matches the inhibition observed in pyramidal cells after their initial excitation in the NPY suppression experiments. The median and 25th and 75th percentile of the data are plotted in the PSTHs.

N: The proposed model of how pyramidal cell inhibition is generated by NPY cells by feedforward inhibition.

O: The proposed model of how pyramidal cell inhibition is generated by feedback inhibition from FS-PV cells, following the suppression of NPY cells.

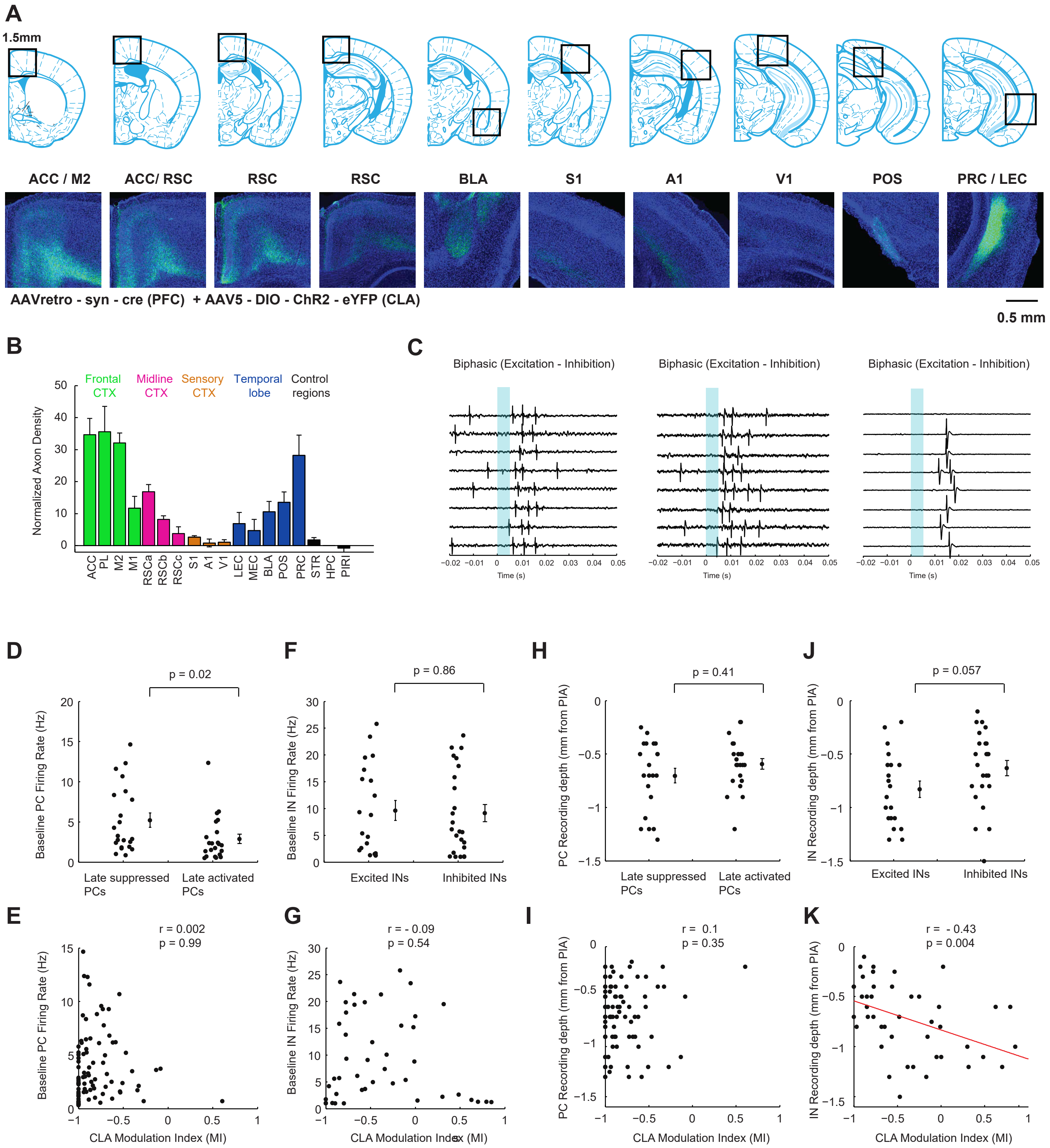

Figure S1. Related to Figure 1.

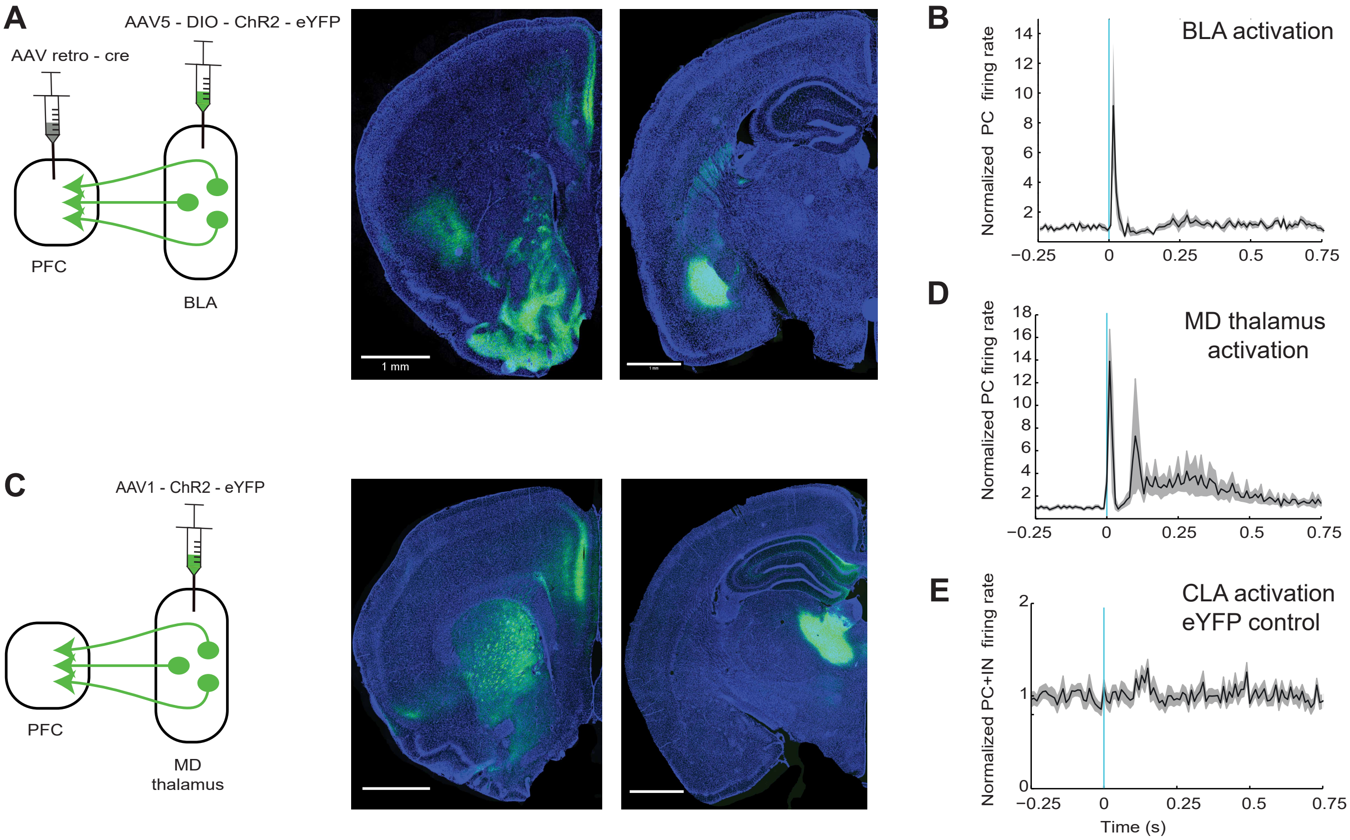

Figure S2. Related to Figure 1.

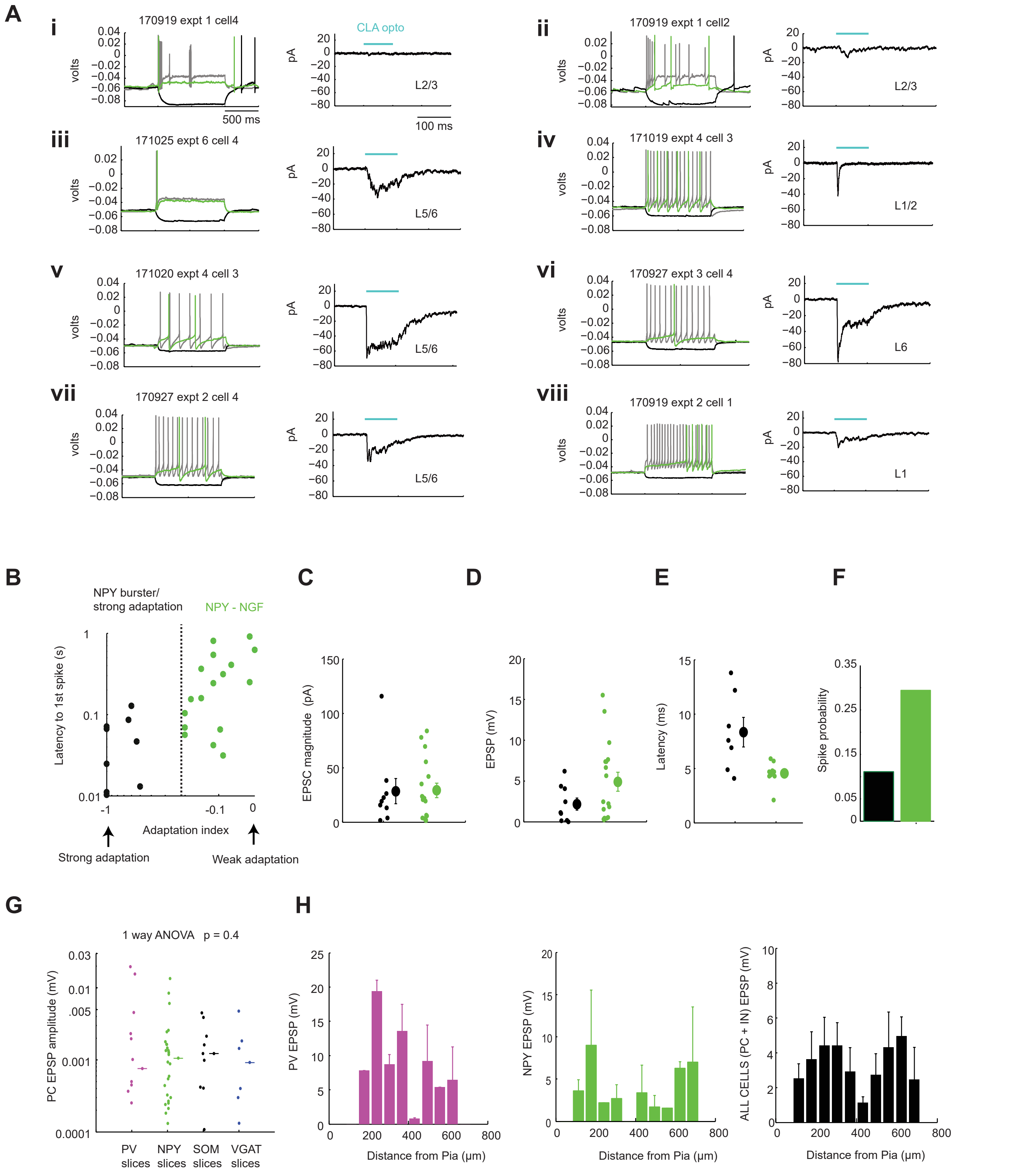

Figure S3. Related to Figure 2 and 3.

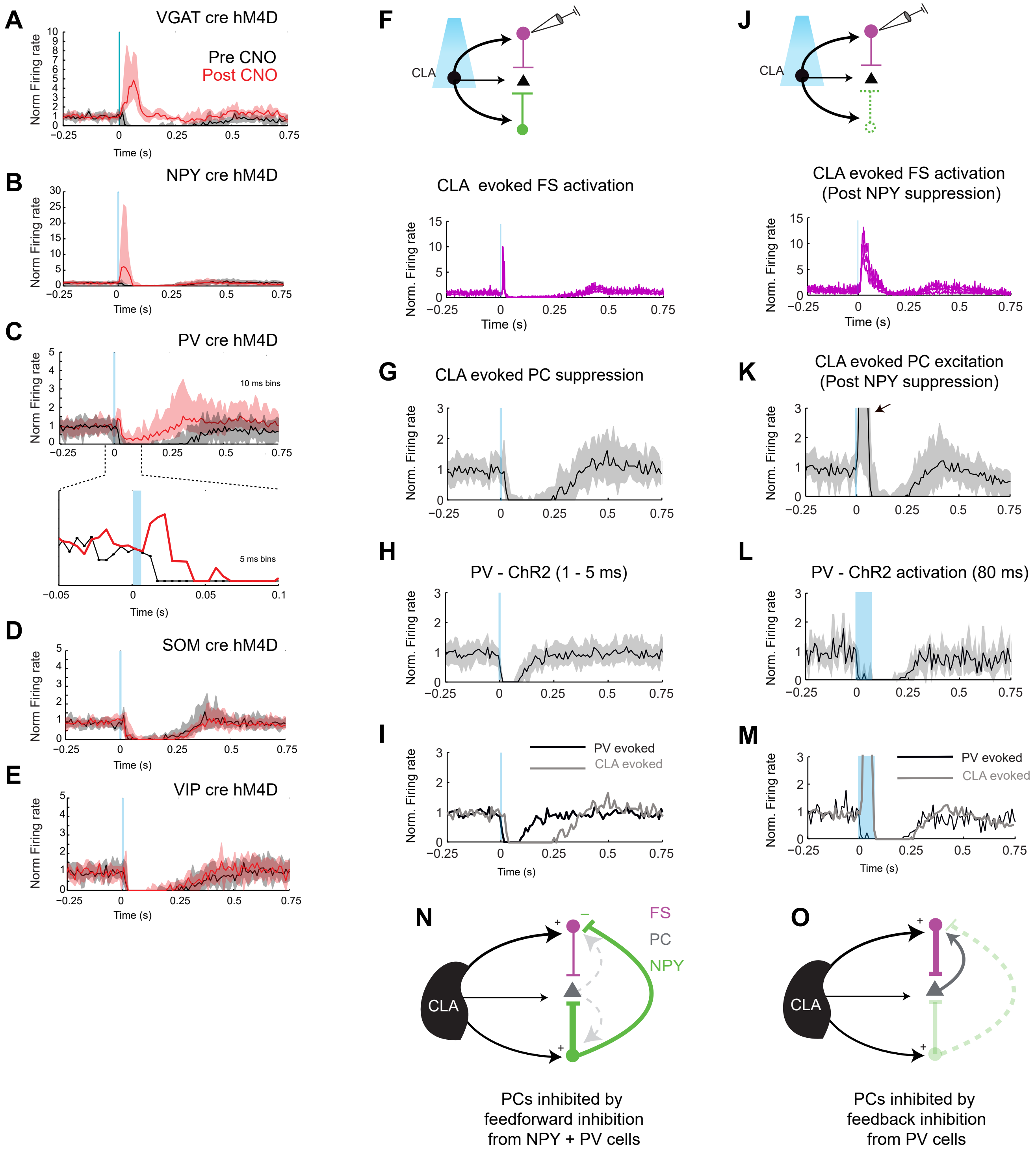

Figure S4. Related to Figure 4.
