# Supplementary material for: Inhibitory Control of Prefrontal Cortex by the Claustrum
Source: Neuron. 2018 Sep 5;99(5):1029–1039.e4. doi: 10.1016/j.neuron.2018.07.031 (PMC6168643; doi:10.1016/j.neuron.2018.07.031)
Supplement: Document S2. Article plus Supplemental Information [file mmc2.pdf]

Neuron

Article

Inhibitory Control of Prefrontal Cortex by the Claustrum

Graphical Abstract

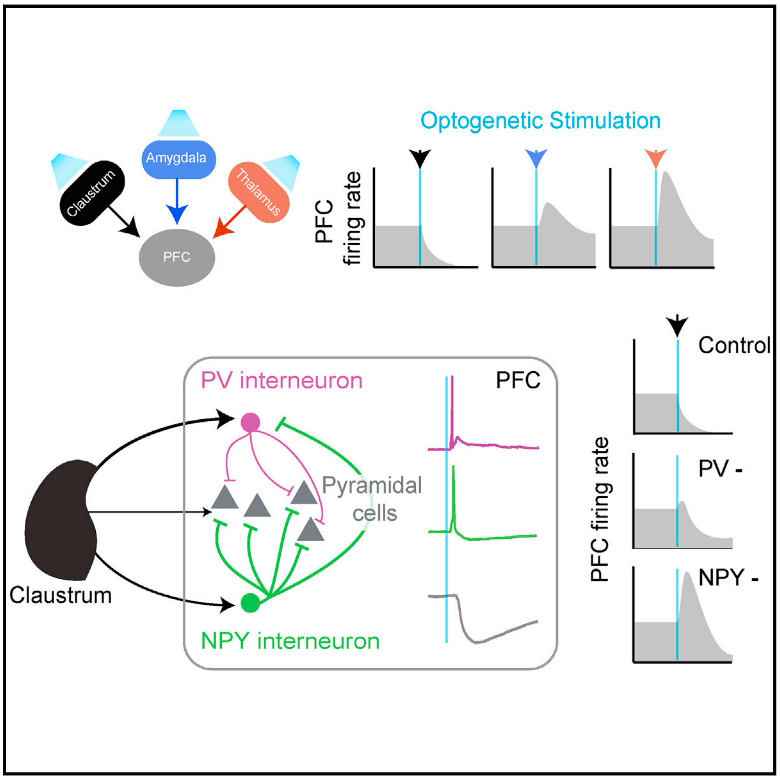

Highlights

- The claustrum strongly inhibits, rather than excites, prefrontal cortex
- Prefrontal NPY and PV interneurons are activated by the claustrum
- Claustrocortical feedforward inhibition is predominantly mediated by NPY neurons

Authors

Jesse Jackson, Mahesh M. Karnani, Boris V. Zemelman, Denis Burdakov, Albert K. Lee

Correspondence

jackson4@ualberta.ca (J.J.), leea@janelia.hhmi.org (A.K.L.)

In Brief

The claustrum provides a dense synaptic input to the cortex, but how claustrocortical projections modulate cortical activity is not known. Jackson et al. show that claustrocortical connections serve to inhibit cortical neural networks by activating specific subtypes of cortical interneurons.

Jackson et al., 2018, Neuron 99, 1029–1039
September 5, 2018 © 2018 Elsevier Inc.
https://doi.org/10.1016/j.neuron.2018.07.031

Inhibitory Control of Prefrontal Cortex by the Claustrum

Jesse Jackson,[1,4,*] Mahesh M. Karnani,[2] Boris V. Zemelman,[3] Denis Burdakov,[2] and Albert K. Lee[1,5,*]
[1]Janelia Research Campus, Howard Hughes Medical Institute, 19700 Helix Drive, Ashburn, VA 20147, USA
[2]The Francis Crick Institute, 1 Midland Road, London NW1 1AT, UK
[3]Department of Neuroscience, Center for Learning and Memory, University of Texas, University Station, C7000 Austin, TX 78712, USA
[4]Department of Physiology, Faculty of Medicine and Dentistry, University of Alberta, Edmonton, AB T6G 2H7, Canada
[5]Lead Contact
*Correspondence: jackson4@ualberta.ca (J.J.), leea@janelia.hhmi.org (A.K.L.)
https://doi.org/10.1016/j.neuron.2018.07.031

SUMMARY

The claustrum is a small subcortical nucleus that has extensive excitatory connections with many cortical areas. While the anatomical connectivity from the claustrum to the cortex has been studied intensively, the physiological effect and underlying circuit mechanisms of claustrocortical communication remain elusive. Here we show that the claustrum provides strong, widespread, and long-lasting feedforward inhibition of the prefrontal cortex (PFC) sufficient to silence ongoing neural activity. This claustrocortical feedforward inhibition was predominantly mediated by interneurons containing neuropeptide Y, and to a lesser extent those containing parvalbumin. Therefore, in contrast to other long-range excitatory inputs to the PFC, the claustrocortical pathway is designed to provide overall inhibition of cortical activity. This unique circuit organization allows the claustrum to rapidly and powerfully suppress cortical networks and suggests a distinct role for the claustrum in regulating cognitive processes in prefrontal circuits.

INTRODUCTION

The hyperconnected neuroanatomical organization of claustrocortical circuits (Atlan et al., 2017; Kim et al., 2016; Wang et al., 2017; White et al., 2017) has led to intense debate over how the claustrum (CLA) contributes to cortical information processing and brain function (Crick and Koch, 2005; Goll et al., 2015; Mathur, 2014). Theoretical and experimental work has suggested the CLA plays a role in attention (Crick and Koch, 2005; Goll et al., 2015), novelty coding (Kitanishi and Matsuo, 2017), sensorimotor integration (Smith et al., 2012), and stress (Seiriki et al., 2017). A common theme that could relate these various cognitive operations is the involvement of the prefrontal cortex (PFC), and indeed the CLA connects most densely with the PFC (Atlan et al., 2017; Smith et al., 2012; White et al., 2017; Zingg et al., 2014). Given the high degree of connectivity with the PFC, the CLA is likely to play an important role in the cortical control of behavior. However, little is known about how CLA activity impacts the cortex in general or the PFC in particular. Previous studies have shown that the CLA can exert either an excitatory or an inhibitory influence on cortical activity (Cortimiglia et al., 1991; Salerno et al., 1984), leaving an unresolved picture of how the CLA influences cortical processing. While anatomical evidence shows that CLA outputs target both inhibitory and excitatory cells within cortical circuits (da Costa et al., 2010; LeVay, 1986; LeVay and Sherk, 1981), there are no physiological data demonstrating how CLA activity alters the firing dynamics of different cortical cell types. Therefore, both the overall and the specific effects of the CLA on the cortex remain unclear. To address these questions, we investigated the impact of CLA activity on the PFC and the cell-type-specific mechanisms underlying this communication.

RESULTS

In order to specifically label and manipulate CLA cells projecting to the PFC, we injected AAVretro-syn-Cre (Tervo et al., 2016) into the anterior cingulate (ACC), prelimbic (PL), and secondary motor (M2) cortex of wild-type mice or rats. The resulting retrograde transport of Cre into neurons that project to the PFC allowed us to functionally isolate the CLA, because neighboring brain regions, such as the striatum and insula, do not project (or project very weakly) to the PFC (Figure 1A). We then injected AAV5-DIO-ChR2-eYFP into the CLA to target this restricted population of Cre-labeled claustrocortical projection neurons for optogenetic stimulation (Figure 1A). We first examined the topography of CLA axons in the brain. Axons from claustrocortical ChR2-eYFP-labeled neurons were found to densely innervate all layers of the PFC, with a preference for deep layers (Figure 1B). Axon collaterals were found throughout the retrosplenial cortex (RSC), basolateral amygdala (BLA), and perirhinal cortex (PRC), with weaker innervation of sensory cortex (Figure S1). Superficial layers were more densely labeled in midline structures relative to lateral cortical regions such as sensory cortex and PRC, which mainly received inputs to the deep layers (Figure S1). Neurons in the CLA responded robustly to ChR2 stimulation, which drove spikes with 1–2 ms latency *in vitro* and *in vivo* (Figure 1C).

To determine the influence of CLA activity on cortical firing patterns, we recorded from putative pyramidal cells (PCs) and interneurons (INs) in the PFC in awake head-fixed mice during CLA

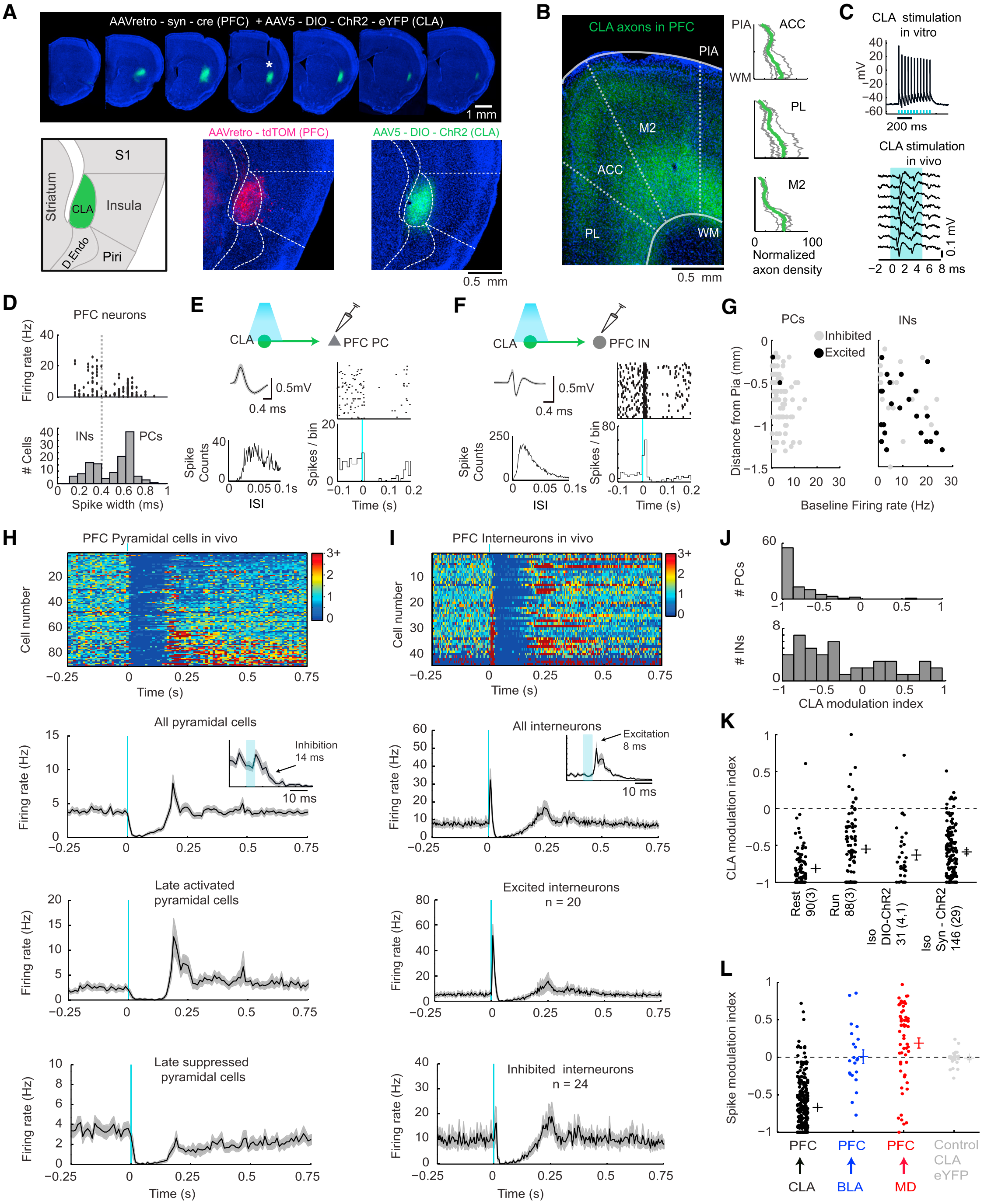

Figure 1. Claustrocortical Projections Control the PFC through Uniquely Strong Feedforward Inhibition *In Vivo*

(A) Top, the distribution of CLA ChR2 virus labeling in the rostro-caudal axis, following injection of AAVretro-syn-Cre into the PFC together with AAV5-DIO-ChR2-eYFP into the CLA. The white asterisk shows the location of the optical fiber. Bottom, a schematic showing the brain regions neighboring the CLA, the retrograde labeling of CLA neurons with tdTomato following injection of AAVretro-CAG-tdTomato in the PFC (red) and ChR2 labeling of claustrocortical neurons (green).

(B) A representative image showing CLA axons in the PFC, and axon density measurements taken in the PFC regions of ACC, PL, and M2. Individual animals (gray) and group mean (green, n = 4) are shown.

(C) Example CLA neurons responding with spiking to 473 nm 5 ms light pulses delivered to the CLA *in vitro* (above) or *in vivo* (below).

(D) A scatterplot of the firing rate versus spike width, and the histogram of spike waveform widths for all (PCs) and interneurons (INs).

(E) An example spike waveform, interspike interval histogram (ISI), and peri-stimulus time histogram (PSTH) for an example PC in response to optogenetic CLA activation (blue line).

(F) The same as in (E), but for an example PFC interneuron.

(legend continued on next page)

stimulation (single 5 ms-long light pulse every 5 s, <10 mW; Figures 1D–1J). The CLA sends excitatory projections to the cortex; thus we expected that activation of this pathway would yield excitation of PCs. However, we detected excitation in only 2/90 PC recordings. CLA activation evoked strong suppression of PC firing rates, and in 33% (30/90) of PC recordings activity was completely silenced (Figures 1E, 1G, 1H, and 1J). Following this initial suppression, many neurons displayed rebound excitation lasting several hundred milliseconds, while others remained suppressed (Figure 1H), and this post-inhibition rebound response was related to the baseline firing rate (Figure S1). In a subpopulation of INs (20/44), CLA activation evoked a biphasic response (excitation–inhibition), whereas the remaining INs (24/44) were inhibited (Figures 1F, 1G, 1I, and 1J), suggesting the activation of inhibitory–inhibitory connections in the cortex. Interneuron excitation occurred with short latency (9.5 ± 0.5 ms, range 7–15 ms) and high fidelity (75% ± 5% of trials, range 16%–100%; Figure S1). Overall, IN modulation index (MI, see STAR Methods) values were significantly negative (−0.38 ± 0.08), as INs were inhibited following excitation (Figures 1I and 1J). INs located in deeper layers were more likely to be excited in accordance with the greater density of CLA axons in deeper layers (Figure S1). Optogenetic stimulation of the CLA during locomotion also reduced PC firing rates, though less than during rest (MI_{rest} = −0.81 ± 0.03; MI_{run} = −0.55 ± 0.05; z = 3.7, p = 1.8 $\times 10^{-4}$, Figure 1K). CLA stimulation also evoked inhibition under anesthesia, or when using a small volume (30 nl) of AAV1-syn-ChR2, which labels all CLA neurons near the injection site irrespective of their projection target (Figure 1K). In control experiments where eYFP was expressed in the CLA without ChR2, no modulation of neural activity was observed (MI = −0.01 ± 0.02; Figure 1L). Therefore, since essentially all excitatory neurons were inhibited, all neurons (PCs and INs) in the PFC appear to be sensitive to CLA inputs through feedforward inhibition (FFI).

PFC inputs from the basolateral amygdala (BLA) and medial dorsal thalamus (MD) also recruit cortical inhibitory circuits (Cruikshank et al., 2012; Delevich et al., 2015; Floresco and Tse, 2007; McGarry and Carter, 2016). To test whether these subcortical inputs to the PFC exert a similarly strong FFI suppression of cortical activity, we optogenetically activated BLA or MD neurons *in vivo* (Figures 1L and S2; see STAR Methods). We found that activation of these other pathways did not yield strong inhibition of PC neural activity. Instead, similar to previous reports (Dilgen et al., 2013; Klavir et al., 2017), we found that activation of the BLA produced excitation in 29% (6/21) of PC recordings, while the other cells were inhibited or not modulated. Activation of MD resulted in excitation in 63% of PCs (36/57), despite the fact that MD inputs are known to evoke strong FFI in the PFC (Cruikshank et al., 2012; Delevich et al., 2015). The CLA produced significantly greater inhibition of PFC activity in awake mice (MI = −0.81 ± 0.03) and anesthetized mice or rats (MI = −0.61 ± 0.07) compared to the BLA (MI = −0.02 ± 0.10) and MD (MI = 0.19 ± 0.07) (Figures 1L and S2). The claustrocortical FFI also appears to be stronger than the direct GABAergic input from the globus pallidus (Saunders et al., 2015). Therefore, compared to other major subcortical inputs, the CLA provides a uniquely strong form of FFI to the PFC.

To study the circuit mechanisms underlying CLA inhibition of the cortex, we performed patch-clamp recordings from PCs and a diversity of molecularly defined INs while stimulating CLA–ChR2 fibers in acute slices from PV-Cre-TOM, SOM-Cre-TOM, VGAT-Cre-TOM, and NPY-hrGFP mice (Figures 2A and 2B; see STAR Methods). CLA fiber activation produced short-latency excitatory currents in PCs, followed by larger inhibitory currents at a delay of 2.0 ± 0.2 ms; Figure 2C). The ratio between the size of excitatory currents (43 ± 20 pA) and inhibitory currents (226 ± 62 pA) ratio was strongly weighted in favor of inhibition (E/I ratio = 0.25 ± 0.07, n = 8). PCs showed strong inhibitory postsynaptic responses that were abolished by ionotropic glutamatergic antagonists, demonstrating CLA inputs evoke inhibitory potentials through local INs (Figure 2D). The excitatory CLA→PC synapses were depressing (adaptation ratio = 0.53 ± 0.17), as measured in response to 20 Hz stimulation (Figure 2E). To determine the cell-type-specific source of the inhibition, we surveyed somatostatin (SOM), fast-spiking parvalbumin (FS-PV), and neuropeptide Y (NPY) interneurons while activating CLA fibers (Figure 2F-L). SOM cells received very weak, nearly absent inputs during CLA fiber stimulation (0.65 ± 0.02 mV, and 3.3 ± 0.6 pA, n = 20; Figures 2F and 2J–2L). In contrast, fast-spiking FS-PV cells were strongly depolarized (10.5 ± 0.2 mV, n = 19), had large excitatory currents (108 ± 35pA), and were driven to spike in 37% of cells at a latency of 5.0 ± 2.5 ms (range, 4.4–15.4 ms) (Figures 2G and 2J–2L). Synaptic responses in PV neurons were also depressing in response to 20 Hz stimulation (adaptation ratio = 0.27 ± 0.05). Next, we used the NPY-hrGFP

(G) The firing rate distribution of PCs and INs as a function of depth measured from the pia. Neurons excited by CLA activation are indicated in black, and inhibited cells are indicated in gray.

(H) Top, the mean normalized firing rate for all PCs in response to optogenetic stimulation of the CLA in awake mice (top). Below, the mean firing rate for all cells, and the subset of cells showing late inhibition or activation following CLA activation. The inset in the mean PSTH shows a magnified view of the group mean PSTH.

(I) Top, the mean normalized firing rate for all INs in response to optogenetic stimulation of the CLA in awake mice (top). Below, the mean firing rate for all INs, and INs which were excited or inhibited. The inset in the mean PSTH shows a magnified view of the group mean PSTH.

(J) Histogram of the single cell modulation index (MI, see STAR Methods) for PCs and INs following the CLA pulse when the animal was at rest.

(K) The comparison of the CLA modulation index for PCs across behavioral states and virus types. Cells are shown during rest, during locomotion (Run), and during isoflurane anesthesia (Iso, with AAV5-DIO-ChR2 or AAV1-syn-ChR2). The values indicate number of cells (and number of animals). In the case of DIO-ChR2, four mice and one rat were used.

(L) The comparison between the strength and direction of PFC modulation by the CLA (n = 37 mice, 267 single-unit and multi-unit recordings), BLA (n = 2 mice, 21 multi-unit recordings), and MD (n = 4 rats, 31 single and multi-unit recordings, and n = 2 mice, 26 single and multi-unit recordings). The data from mice and rats for MD experiments were pooled, as the mean MI for rats (0.21 ± 0.08) was not significantly different than for mice (0.15 ± 0.12, p = 0.6). Control experiments with CLA–eYFP are also shown (n = 2 mice, 24 recordings). Interneuron recordings were excluded from all analyses here except the control recordings. Data are expressed as mean ± SEM. See also Figure S1.

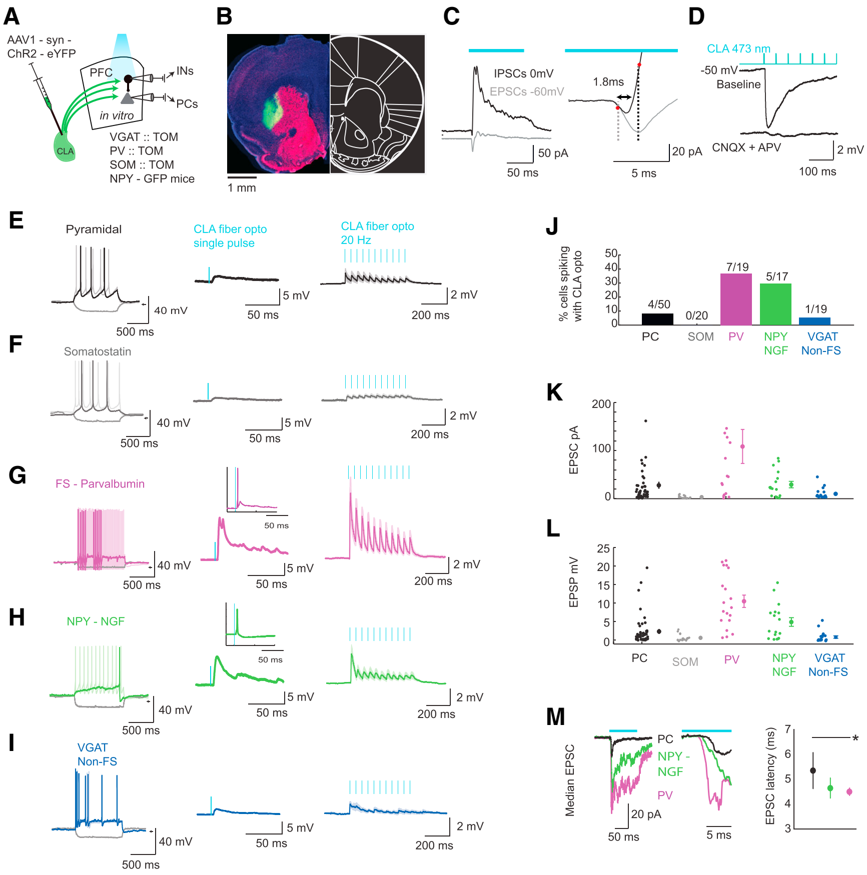

Figure 2. Cortical PV and NPY Inhibitory Neurons Respond Strongly to CLA Activation

(A) A schematic showing the injection of ChR2 into the CLA, followed by whole-cell patch-clamp recordings from INs and PCs in the PFC *in vitro* while blue light was used to activate CLA axon terminals.

(B) An example coronal section showing the localized ChR2 in the CLA.

(C) The mean voltage-clamp response in PCs during optogenetic activation of CLA fibers. n = 5 cells with clear excitatory and inhibitory postsynaptic currents (EPSCs and IPSCs). On the right is a magnified view of the left, highlighting the fast excitation followed by inhibition. Onsets were calculated as the start of the 10%–90% rise times.

(D) Current-clamp responses in an example PC during 20 Hz CLA activation. Responses are shown before and after addition of CNQX and APV. PCs receive FFI from local interneurons, as the inhibitory postsynaptic potential (IPSP) was blocked by antagonists of glutamatergic transmission. The same result was obtained in n = 3 cells.

(E–I) Example whole-cell recordings from a PC, and somatostatin (SOM), fast-spiking parvalbumin (FS-PV), neuropeptide Y neurogliaform cell (NPY–NGF), and VGAT (non-fast spiking) interneurons. Current steps for firing patterns (left) are −30, +40, and +70 pA (PC); −30, +20, and +50 pA (SOM); −30, +20, and +50 pA (PV); −30, +10, and +50 pA (NPY); and −30, +20, +40 pA (VGAT). Darkest lines represent responses to the middle current value. Small horizontal lines indicate −60 mV. The mean response of each cell to a single pulse of light onto the CLA fibers (middle). Insets for FS-PV and NPY-NGF cells show the same cell spiking in response to CLA activation. The average response to 20 Hz stimulation is shown (right).

(J) The proportion of each cell type showing spiking (at least two spikes in ten trials) in response to CLA fiber activation.

(K and L) The EPSC (K) and excitatory postsynaptic potential (EPSP) amplitude (L) for all cells of each subtype.

(M) The latency of EPSC onset for FS-PV, NPY-NGF, and PC neurons. PV and NPY-NGF cells had activation onsets earlier than PCs.

For these data, four VGAT-Cre-TOM, four SOM-Cre-TOM, four PV-Cre-TOM, and six NPY-hrGFP mice were used. In (K), all pairwise differences were significantly different at the $p < 0.05$ level except the VGAT-SOM, PC-NPY, and PV-NPY comparisons. In (L), all pairwise comparisons were significant at the $p < 0.05$ level (Bonferroni-Holm correction), except the difference between SOM and VGAT (non-FS) cells. *$p < 0.05$. Data are expressed as mean ± SEM throughout. See also Figure S3.

mouse line (Chittajallu et al., 2013; Krook-Magnuson et al., 2011; van den Pol et al., 2009) to record the subpopulation of NPY neurons known as neurogliaform (NGF) cells. NGF cells have a strong, widespread inhibitory influence on cortical PCs and INs (Chittajallu et al., 2013; Jiang et al., 2015; Tamás et al., 2003), using slow $GABA_A$-mediated inhibition, that is separable from fast inhibition arising from FS-PV cells (Fuentealba et al., 2008; Overstreet-Wadiche and McBain, 2015; Szabadics et al., 2007). We identified putative NPY-NGF cells as those with weakly adapting spike trains in response to supra-threshold current injection (Karagiannis et al., 2009; Kawaguchi and Kubota, 1997; Overstreet-Wadiche and McBain, 2015; Simon et al., 2005; Tamás et al., 2003; Tricoire et al., 2010) (Figure S3). This basic categorization yielded 17/26 NPY cells, 11 of which had the classic NGF phenotype showing late spiking in response to "just past threshold" depolarization (Figures 2H and S3). These NPY-NGF cells were also strongly depolarized by CLA inputs (4.9 ± 1.1mV), had moderate-sized excitatory current (29 ± 6pA), had depressing responses to 20 Hz pulse trains (adaptation ratio = 0.27 ± 0.10), and showed CLA-evoked action potentials in 29% of cells at a latency of 9.8 ± 1.5ms (range, 6.7–15.6 ms) (Figures 2H and 2J–2L). We also sampled interneurons in the VGAT-Cre-TOM mouse (labeling all interneurons) to investigate the possibility that interneurons not specifically labeled in other experiments would be excited by CLA inputs. When we excluded clear FS cells (<0.2 ms spike width, peak firing rate >100 Hz), the magnitude of responses in these randomly sampled interneurons was small (EPSP = 0.96 ± 0.32 mV; EPSC = 7.3 ± 2.6 pA, n = 19) (Figures 2I–2L). Therefore, FS-PV and NPY cells were the two main classes of interneurons depolarized by CLA inputs. The latency to optical activation was shorter in NPY-NGF cells (4.5 ± 0.3 ms) and FS-PV cells (4.6 ± 0.2 ms) than in PCs (6.4 ± 0.7 ms) (Figure 2M), suggesting the postsynaptic AMPA receptor composition is different between INs and PCs as in the thalamocortical system (Cruikshank et al., 2007; Hull et al., 2009). The smaller and slower kinetics

CellPress

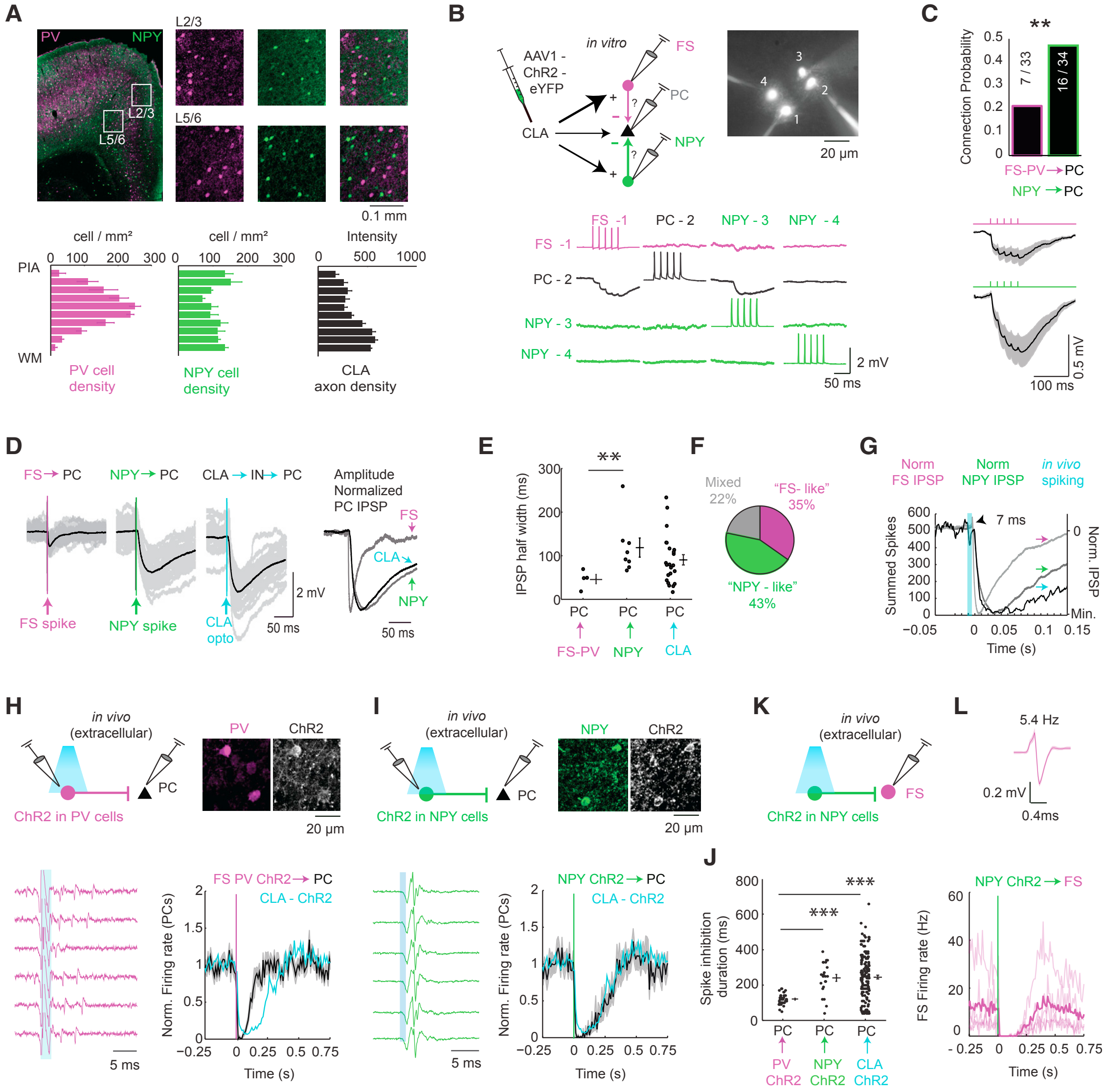

Figure 3. The Comparison between NPY- and PV-Mediated Inhibition in the PFC

(A) Anatomical characterization of the layer dependence of PV and NPY neurons in the PFC. An example image is shown. Areas denoted by the white boxes are expanded on the right. The histograms below show the mean ± SEM of the cell density as a function of the normalized distance from the pia to white matter (WM). The density of CLA axons as a function of distance from pia is also shown. Densities were averaged across the prelimbic cortex, anterior cingulate cortex, and secondary motor cortex (n = 4 mice). PV cells were identified immunohistochemically (magenta) in NPY-GFP mice.

(B) A schematic showing the comparison between feedforward inhibition arising from either NPY or FS cells *in vitro*. Patch-clamp recordings were made from FS-PV cells, NPY cells, and PCs. An example quadruple-patch experiment where a FS, PC, and two NPY cells were simultaneously recorded, and the connectivity between cells, is shown during 50 Hz pulse trains.

(C) The connection probability for pairs of FS cells and PCs and for NPY cells and PCs. The mean IPSP in response to FS or NPY stimulation is shown below.

(D) An example experiment showing the unitary monosynaptic IPSP from an FS cell to PC, from a NPY cell to PC, and the IPSP in response to optogenetic CLA stimulation. Gray traces throughout are individual trials. For unitary connections, one spike was elicited in the presynaptic cell. The amplitude-normalized responses are shown on the right. Note the similarity between the NPY→PC IPSP and the CLA-mediated IPSP in this particular experiment.

(E) The decay time half-widths for IPSPs elicited by FS cells, by NPY cells, and by CLA stimulation. The CLA inhibitory kinetics span a range of values *in vitro*. However, the CLA-mediated IPSP half-width was more similar to the NPY than FS values, and NPY-mediated IPSPs were slower than FS-mediated IPSPs.

(F) The pie chart shows the proportion of CLA-evoked IPSPs that were classified as "NPY-like" or "FS-like," or mixed based on IPSP rise times and IPSP half-widths.

(G) FS and NPY IPSPs overlaid on the *in vivo* spiking response to CLA stimulation. IPSPs were shifted to 7 ms to match the predicted onset of IPSPs *in vivo* based on the observation that interneurons began spiking ~7 ms following CLA stimulation.

(H) *In vivo* optogenetic activation of PV interneurons suppresses PC neurons. An example putative opto-tagged PV neuron in the PFC responding to 2 ms, 473 nm light pulses. The cell fired in one to three spike bursts similar to many interneurons recorded during CLA activation (Figure S1). On the right is the mean response of PCs to PV activation (black) and the mean response in all cells inhibited by CLA activation (blue). Dark lines show the mean, and shaded regions show the SEM (n = 3 mice, 34 PC recordings).

(legend continued on next page)

of excitatory responses in PCs helps explain why they rarely fire action potentials in response to CLA activation *in vivo*, as their excitation would be quenched by the more rapidly acting inhibition. Collectively, these experiments show that two main types of interneurons—PV and NPY—are responsible for claustrocortical FFI.

Interneurons containing NPY and PV are by and large non-overlapping cell types with distinct layer distributions in the PFC (Figure 3A). PV cells are mainly located in layer 5, while NPY cells are distributed throughout all cortical layers (Xu et al., 2010). We compared the CLA-evoked disynaptic IPSP kinetics in PCs with FS→PC and NPY→PC kinetics to determine if one GABAergic subtype may be more responsible for CLA-controlled FFI (Figures 3B–3G). To begin with, the NPY→PC connection probability (16/34 pairs synaptically connected) was greater compared to FS-PV→PC (7/33 connected, Figure 3C), suggesting that NPY cells have a stronger influence on PCs (Jiang et al., 2015). Moreover, NPY-NGF cells provide slow $GABA_A$ and $GABA_B$-mediated inhibition in the cortex and have slower kinetics than fast $GABA_A$ receptors activated by FS-PV cells (Banks et al., 2000; Capogna and Pearce, 2011; Szabadics et al., 2007; Tamás et al., 2003). We confirmed that FS→PC synapses have shorter IPSP rise times (5.6 ± 1.1 versus 22.3 ± 3.5 ms; p = 0.004) and decay half-widths (46.3 ± 10.6 versus 118.6 ± 21.9 ms, p = 0.008; Figures 3D–3F) than NPY→PC IPSPs. In many cases, the CLA-evoked IPSP kinetics in PCs matched those of the NPY→PC synapse (43%, 10/23), implying NPY cells were determining FFI in these cells (Figure 3E). In other cases, the CLA-evoked IPSP kinetics in PCs fit the dynamics of FS→PC synapses (35%). In the remainder of cases we observed a fast IPSP rise time with a slow decay suggesting co-innervation from both FS and NPY cells (22%, Figure 3F), and averaged across all cells the decay time of CLA mediated IPSPs (90 ± 12 ms) was better fit by the slower NPY–NGF inhibition. Finally, *in vivo*, the kinetics of spike suppression followed a time course more similar to NPY-PC IPSPs (Figure 3G), again suggesting the CLA-to-NPY interneuron circuit could play a special role in the strong inhibition seen in awake mice. To test the *in vivo* inhibitory drive from PV or NPY interneurons to PCs, we directly activated each interneuron type using ChR2 in PV-Cre and NPY-Cre mice and compared the dynamics of PC spike inhibition with CLA activation (Figures 3H–3L). Brief (1–5 ms) activation of PV or NPY neurons elicited powerful suppression of PC firing (Figures 3H and 3I). However, the spike inhibition recovery with PV activation was significantly faster (119 ± 5 ms) than with NPY activation (240 ± 19 ms, z = 4.64, $p = 3.5 \times 10^{-6}$) or CLA activation (244 ± 10 ms; z = 5.38, $p = 7.8 \times 10^{-8}$). The recovery from inhibition during CLA or NPY activation was not different (z = 0.02, p = 0.98; Figure 3J). Therefore, CLA-mediated inhibition cannot be explained by PV-mediated inhibition alone, and appears to be better correlated with the dynamics of NPY-mediated inhibition. In addition, NPY activation also potently suppressed FS firing *in vivo* (n = 5/5 cells; Figures 3K and 3L), confirming the presence of NPY-to-FS-mediated inhibition described previously *in vitro* (Jiang et al., 2015).

To test the role of different interneuron subtypes in claustrocortical FFI *in vivo*, we activated the CLA using ChR2 while pharmacogenetically suppressing specific interneuron subclasses using the corresponding Cre mice with AAV5-CAG-FLEX-hM4D injected into the PFC (Figures 4A–4I). CLA modulation of PFC neurons was compared pre- and post-clozapine–N–oxide (CNO) administration, which induces hyperpolarization and reduces the probability of synaptic release in Cre-hM4D cells (Stachniak et al., 2014; Sternson and Roth, 2014). As the claustrocortical projection is excitatory, we predicted that suppressing interneurons would reduce FFI and unmask claustrocortical excitation. First, the suppression of all GABAergic interneurons in VGAT-Cre-hM4D mice converted claustrocortical inhibition to excitation (MI_{pre}, −0.55 ± 0.1; MI_{post}, 0.58 ± 0.08, z = 3.8, $p = 1.5 \times 10^{-4}$), demonstrating that the direct excitatory CLA inputs were sufficient to drive PC bursts in the cortex (Figures 4G and 4H and S4). Next, suppression of PV interneurons led to an overall reduction of FFI following CLA activation (MI_{pre}, −0.65 ± 0.05; MI_{post}, −0.19 ± 0.04; z = 5.7, $p = 9.39 \times 10^{-9}$; Figures 4D and 4H). However, only 7% of the PC recordings showed short latency (5–30 ms) excitation following PV suppression, and robust inhibition was still observed (Figures 4G and 4H), suggesting PV interneurons are not the major contributor to the suppression of excitation in the claustrocortical circuit. The pharmacogenetic suppression of NPY interneurons converted claustrocortical inhibition to excitation (MI_{pre}, −0.48 ± 0.05; MI_{post}, 0.51 ± 0.08; z = 6.8, $p = 8.3 \times 10^{-12}$; Figures 4E, 4G, and 4H), a change that was significantly greater than in PV cell-suppression experiments (z = 5.8, $p = 7.3 \times 10^{-9}$). These data suggest that NPY cells are responsible for suppressing short latency excitation of PCs through FFI. Following the increase in PC activity during NPY suppression, there was delayed inhibition that occurred concomitantly with an increase in FS interneuron activity (Figures 4E and S4). During NPY suppression, FS cells showed an increased CLA modulation index (baseline = −0.63 ± 0.13; NPY suppression = 0.58 ± 0.11; z = 3.25, p = 0.001), an increase in maximum firing rate (baseline = 15.5 ± 4.4 Hz; NPY suppression = 27.9 ± 5.6; z = 2.01, p = 0.04), and an increased proportion of cells excited by CLA

(I) *In vivo* optogenetic activation of NPY interneurons suppresses PC neurons. An example putative opto-tagged NPY neuron in the PFC responding to 1 ms of 473 nm light pulses. The cell fired one spike per trial. On the right is the mean response of PCs to NPY activation (black) and the mean response in all cells inhibited by CLA activation (blue). Dark lines show the mean and shaded regions the SEM (n = 2 mice, 22 PC recordings).

(J) The duration of spike inhibition in PV-ChR2, NPY-ChR2, and CLA activation experiments. Spike inhibition duration was measured by the time elapsed between falling below 50% of the baseline firing rate and recovering back to 50% of the baseline firing (n = 32 mice; 155 recordings for CLA activation).

(K) Schematic depicting the experiment in which NPY cells are activated with ChR2 while recording from putative FS interneurons.

(L) The spike shape of an extracellular recording of a putative FS cell (above) that was identified during NPY-ChR2 activation. The baseline firing rate of this neuron is indicated above the waveform. The PSTHs for all experiments are shown below (n = 5). The dark line is the mean, and light shaded lines are individual experiments. Data are expressed as mean ± SEM throughout.

**p < 0.01, ***p < 0.001. See also Figure S3.

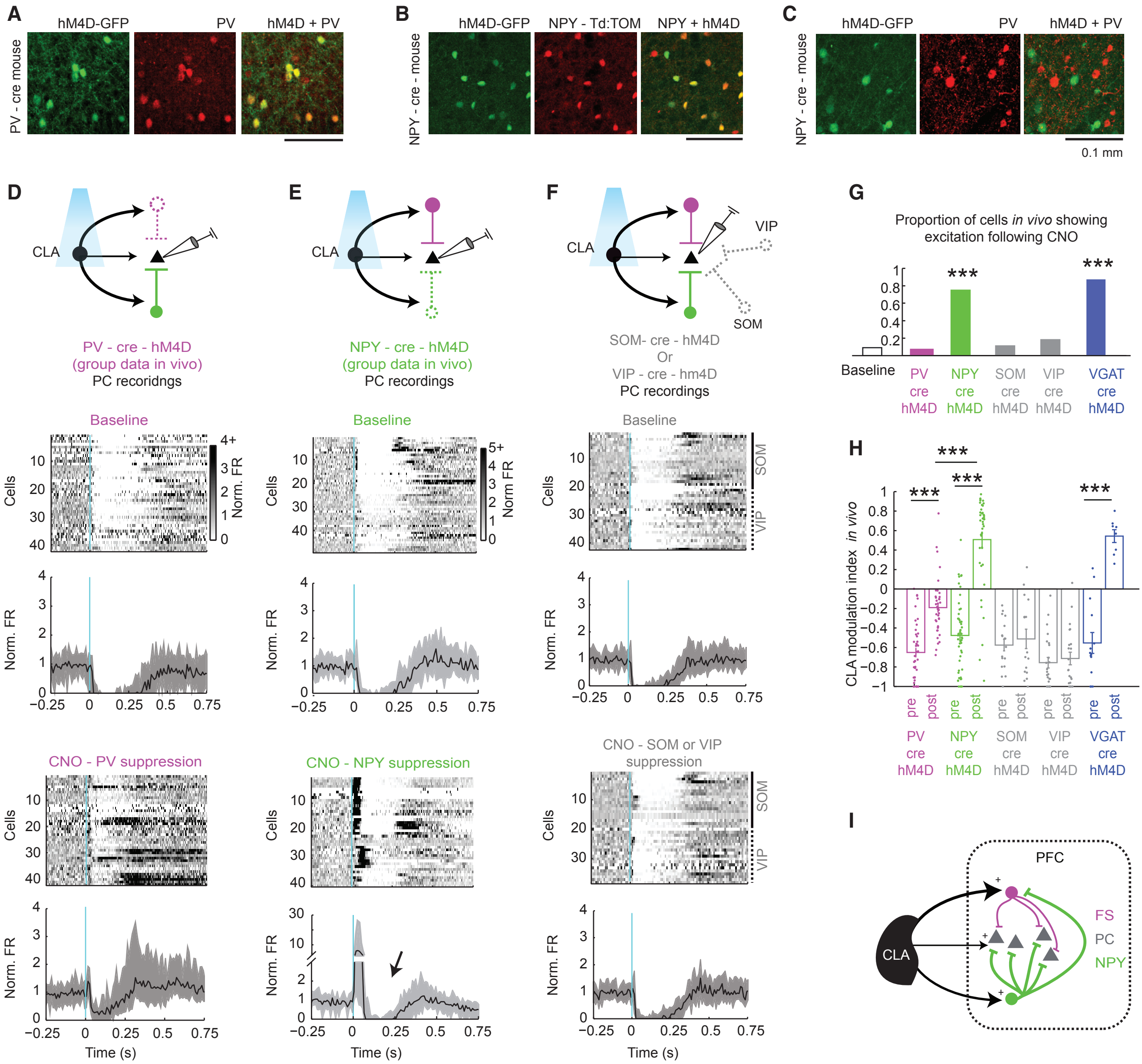

Figure 4. NPY Cells Control Claustrocortical Feedforward Inhibition in the PFC

(A) An example image showing the localization of the AAV5-FLEX-hM4D receptor in PV cells in the PFC in PV-Cre mice.

(B) An example image showing the localization of the AAV5-FLEX-hM4D receptor in NPY cells in the PFC in NPY-Cre-td-Tomato mice.

(C) An example image showing the lack of localization of the hM4D receptor in PV cells when injected into NPY-Cre mice. PV cells were revealed using immunohistochemistry.

(D) A schematic showing the experiment in which PCs were recorded during optogenetic CLA stimulation before and after the suppression of PV cells (top). The normalized firing rate (Norm FR) of all PCs measured *in vivo* is shown during baseline (middle) and post-CNO (bottom). CLA activation (5 ms) occurred at 0 s. Mice were acutely anesthetized with light (0.9%–1.0%) isoflurane.

(E) A schematic showing the experiment in which PCs were recorded during optogenetic CLA stimulation before and after the suppression of NPY cells (top) under the same conditions as in (D). The response of all PCs measured *in vivo* is shown during baseline (middle) and post-CNO (bottom). FS cells also increased their firing rate and duration of bursting following NPY suppression (Figure S4). Therefore, the inhibition occurring after PC excitation is proposed to arise from feedback inhibition from FS interneurons (arrow, also see Figure S4).

(F) A schematic showing the experiment in which PCs were recorded during optogenetic CLA stimulation before and after the suppression of SOM or VIP cells in different experiments (top). The response of all PCs measured *in vivo* is shown during baseline (middle) and post-CNO (bottom). The individual experiments in SOM-Cre or VIP-Cre mice are indicated on the right side of the cell raster.

(G) The proportion of experiments in which PC excitation was detected following the suppression of each interneuron type (for all group PSTHs, see Figure S4).

(legend continued on next page)

activation (baseline = 50%, n = 8/16; NPY suppression = 78%, n = 7/9). When we optogenetically activated FS-PV cells with long trains of light (80 ms) mimicking the increased duration of FS activity, PCs were inhibited in a manner similar to the late inhibition in NPY suppression experiments (Figure S4). Therefore, this late inhibition remaining following NPY blockade is most likely explained not by direct feedforward CLA-mediated effects but by feedback inhibition from local circuit interneurons that increase their CLA evoked activity in the absence of NPY activity. Similar experiments suppressing SOM interneurons (MI_{pre}, −0.58 ± 0.06; MI_{post}, −0.51 ± 0.10; z = 0.3, p = 0.9) or VIP interneurons (MI_{pre}, −0.76 ± 0.05; MI_{post}, −0.71 ± 0.06; z = 0.3, p = 0.8) did not alter claustrocortical FFI (Figures 4F–4H). These VIP-Cre-hM4D and SOM-Cre-hM4D experiments also demonstrate that any nonspecific actions of CNO itself (Gomez et al., 2017) are not responsible for changes in inhibition. Although NPY neurons can express VIP and SOM (Karagiannis et al., 2009; Kawaguchi and Kubota, 1997), the fact that suppression of these other cell types did not change claustrocortical FFI suggests the effects we describe here do not arise from VIP or SOM interneurons.

DISCUSSION

The CLA is connected to most cortical regions, yet very little was known about the physiology of claustrocortical connections. To explore how CLA activity affects cortical networks, we used pathway-specific optogenetic activation to show that claustrocortical projections drive robust feedforward inhibition (FFI) of excitatory pyramidal cells (PCs) within the PFC. Although many structures provide long-range FFI to PFC networks (Cruikshank et al., 2012; Delevich et al., 2015; Dilgen et al., 2013; Floresco and Tse, 2007; Gabbott et al., 2006; McGarry and Carter, 2016), the efficacy of the claustrocortical projection for the suppression of cortical activity appears to be stronger than other afferents. This is likely due to several unique features of the claustrocortical circuit. First, we found that the strength of CLA excitation onto PV and NPY interneurons (INs) is stronger than onto neighboring excitatory pyramidal cells (PCs). Studies describing the ratio of excitation to feedforward inhibition from the BLA and MD have shown that these inputs evoke an excitation/inhibition ratio of ∼1 for BLA (McGarry and Carter, 2016) and 0.76 for MD (Collins et al., 2018), whereas with the CLA input we observed a reduced ratio of 0.25. Therefore, the CLA inputs evoke an imbalanced and excess level of FFI, which would favor net inhibition. Second, we found that the excitatory dynamics of CLA synapses onto INs were faster than onto PCs, preventing them from reaching spike threshold. Such fast FFI also arises in the thalamocortical pathway (Cruikshank et al., 2007), but there the excitatory inputs to PCs are convergent enough to allow them to fire action potentials in response to input *in vivo* (Bruno and Sakmann, 2006; Gabernet et al., 2005). Third, we demonstrated that the claustrocortical FFI is highly dependent on NPY INs, whereas thalamocortical FFI is thought to depend predominantly on FS-PV cells (Bruno, 2011; Cruikshank et al., 2007; Delevich et al., 2015; Gabernet et al., 2005). These differences in strength, timing, convergence of excitation, and postsynaptic cell types may differentiate the claustrocortical pathway from the thalamic and BLA inputs.

Previous work on the physiology of claustrocortical projections was carried out using electrical stimulating electrodes (Cortimiglia et al., 1991; Salerno et al., 1984, 1989). Given that the CLA is situated adjacent to the external capsule, off-target and/or antidromic effects cannot be entirely ruled out when interpreting the results of these early experiments. In addition, this prior work was performed under deep anesthesia, which can alter neurotransmission (Nishikawa and MacIver, 2001). These reports found that CLA stimulation could result in either inhibition or excitation followed by inhibition. Our approach circumvents the caveats associated with electrical stimulation by specifically activating the cell bodies of CLA neurons projecting to the PFC. With this approach, we observed a near-uniform inhibition of PC spiking in response to CLA activation. Interestingly, in a human patient, unconsciousness was evoked by stimulation of the CLA (Koubeissi et al., 2014). Our data suggest that the loss of consciousness may arise from the inhibition of cortex. The use of optogenetics here does allow for specific activation of CLA output neurons; however, further *in vivo* recording data are required from these neurons to determine the endogenous firing patterns of CLA circuits. Work in primates has shown that CLA neurons can display brief 30–40 Hz bursts lasting 50–200 ms following sensory stimulation (Remedios et al., 2010). Therefore, although the optogenetic activation is artificial, it may mimic the large increase in CLA activity in some natural contexts. Future work performing dual CLA–PFC recordings will enable the assessment of how the dynamics of natural CLA activity correlates with ongoing context dependent PFC activity.

Our results show that the CLA functions as an inhibitory brake on the output of the prefrontal cortex, providing a uniquely strong FFI input via NPY and PV INs. How might this "blanket-like" inhibition contribute to the representation and processing of information in the PFC? One possibility is that CLA inputs provide a large-scale form of lateral inhibition, whereby context-specific populations of CLA neurons suppress designated cortical regions, while leaving others free to encode stimuli according to specific behavioral demands. In support of this idea, a recent study has shown that suppression of CLA renders mice unable to ignore distracting sensory stimuli (Atlan et al., 2018). In this work, mice undergoing CLA suppression performed normally on attention-based tasks and were only impaired when they were required to ignore irrelevant auditory stimuli. Our work suggests that the suppression of extraneous stimuli may be accomplished by the CLA driving cortical NPY cells. Another

(H) The CLA modulation index from all recordings, pre- and post-CNO in different interneuron suppression experiments. PV-cre (n = 10 mice), NPY-Cre (n = 7 mice), SOM-Cre (n = 4 mice), VIP-Cre (n = 5 mice), and VGAT-Cre (n = 3 mice).

(I) A schematic model of claustrocortical FFI based on the results presented here. CLA evokes weak excitation in PCs and stronger excitation in FS-PV cells and NPY cells. Arrow width indicates the relative strength of each connection.

The median and 25th and 75th percentile of the data are plotted in the PSTHs (D–F), and mean ± SEM are shown in (H). ***p < 0.001. See also Figure S4.

recent study has shown that cortical inputs to the CLA also evoke strong FFI within the CLA itself (Kim et al., 2016), suggesting that the cortex may select the CLA circuit to activate, and this corticoclaustral selection may then dictate which cortical ensemble to suppress. The CLA may also serve a more generalized function by dampening background activity rates and reducing network noise, ensuring that excitatory inputs from other pathways, such as the MD or BLA, can activate the appropriate PFC ensemble required in a particular behavioral context.

Feedforward inhibition is a fundamental feature of many cortical circuits (Isaacson and Scanziani, 2011). In the thalamocortical system, fast-spiking PV cells are thought to be the critical interneuron class responsible for FFI (Cruikshank et al., 2007, 2012; Delevich et al., 2015). Surprisingly, we found that suppressing PV cells did not lead to much excitation with claustrocortical stimulation. Rather, the NPY interneuron class appears to be largely responsible for claustrocortical inhibition. In the cortex, NPY cells include the class of interneuron known as neurogliaform cells, which are known to elicit slow forms of inhibition using slow $GABA_A$ and $GABA_B$ receptors (Capogna, 2011; Szabadics et al., 2007; Tamás et al., 2003). Given that NPY cells provide inhibitory modulation of all other cell types, especially PV cells and PCs (Chittajallu et al., 2013; Jiang et al., 2015; Oláh et al., 2009; Szabadics et al., 2007; Tamás et al., 2003), it is perhaps not surprising that these cells can exert such strong modulation of cortical circuits. Although PV cell suppression only modestly reduced claustrocortical FFI, these interneurons clearly play a role in this circuit. It is possible that PV cells relay CLA signals to a specific subset of cortical PCs, whereas NPY cells have a more global role in cortical inhibition, or different PC networks may be controlled by CLA→NPY→PC and CLA→PV→PC circuits, or the two interneurons types may act synergistically to control PC networks. While much remains to be learned about how NPY interneurons control activity within neural circuits, our data provide evidence that the CLA plays an important role in modulating these cells in the cortex. Although we identify NPY and PV cells as mediators of CLA-evoked FFI, other less-well-studied interneuron populations may also be activated by CLA inputs. For example, PFC chandelier cells (ChC) inhibit the firing of PCs (Lu et al., 2017). However, these interneurons only inhibit a subpopulation of PCs and on average elicit weaker inhibition than that observed with CLA activation; therefore, it is unlikely that these cells play a dominant role in CLA-mediated FFI. Although PV, NPY, SOM, and VIP interneurons comprise the vast majority of all cortical interneurons, there are other interneuron classes that were not targeted in our slice recording and interneuron suppression experiments. Future work using more refined transgenic mouse lines will be required to explicitly test if and how strongly these interneuron subpopulations are activated by the CLA.

The balance between excitation and inhibition in the PFC is critical for a wide range of neural processes and behaviors such as reward (Otis et al., 2017), fear/anxiety signaling (Courtin et al., 2014; Do-Monte et al., 2015; Likhtik et al., 2014; Tovote et al., 2015), and social behaviors (Felix-Ortiz et al., 2016; Gunaydin et al., 2014; Yizhar et al., 2011). NPY cells in the cortex are active during slow-wave sleep (Gerashchenko et al., 2008) and UP-states (Neske et al., 2015), and they help prevent seizure generation during these "offline" states (Hall et al., 2015). Given the strong modulation of PFC neural networks by the CLA, future investigation should seek to understand how claustrocortical FFI facilitates information processing in these different brain states and behavioral contexts.

STAR★METHODS

Detailed methods are provided in the online version of this paper and include the following:

- KEY RESOURCES TABLE
- CONTACT FOR REAGENT AND RESOURCE SHARING
- EXPERIMENTAL MODELS AND SUBJECT DETAILS
- METHOD DETAILS
  - Viruses and surgery
  - *In vivo* physiology
  - Optogenetic activation
  - *In vitro* slice preparation and recording
  - Immunohistochemistry
  - Analysis of axon density
  - Analysis of electrophysiology data
- QUANTIFICATION AND STATISTICAL ANALYSIS
- DATA AND SOFTWARE AVAILABLILTY

SUPPLEMENTAL INFORMATION

Supplemental Information includes four figures and can be found with this article at https://doi.org/10.1016/j.neuron.2018.07.031.

ACKNOWLEDGMENTS

We thank Salvatore DiLisio for assistance with virus injections; Monique Copeland with histology; Sarah Erwin for animal care; Steve Sawtelle and Jon Arnold for help with the design and construction of the mouse treadmill; Jim Cox and Amanda Zeladonis for facilitating collaboration between The Crick and Janelia; Janelia Virus Services for producing viruses; Joshua Dudman, Adam Hantman, and Jeremy Cohen for comments on the manuscript; and Laura Hart for administrative assistance. The work was supported by the Howard Hughes Medical Institute (J.J. and A.K.L.); The Francis Crick Institute, which receives its core funding from Cancer Research UK, the UK Medical Research Council, and the Wellcome Trust (D.B.); and a European Union Horizon 2020 Research and innovation program Marie Skłodowska-Curie grant (M.M.K.). This work was supported by The Francis Crick Institute, which receives its core funding from Cancer Research UK (FC001055), the United Kingdom Medical Research Council (FC001055), and the Wellcome Trust (FC001055).

AUTHOR CONTRIBUTIONS

Conceptualization, J.J. and A.KL.; Methodology, J.J. and M.M.K.; Investigation, J.J. and M.M.K.; Writing – Original Draft, J.J. and A.K.L.; Writing – Review & Editing, J.J., M.M.K., B.V.Z., D.B., and A.K.L..; Funding Acquisition, D.B. and A.K.L.; Resources, D.B., B.V.Z., and A.K.L.; Supervision, A.K.L.

DECLARATION OF INTERESTS

The authors declare no competing financial interests.

Received: January 16, 2018
Revised: June 17, 2018
Accepted: July 18, 2018
Published: August 16, 2018

REFERENCES

Atlan, G., Terem, A., Peretz-Rivlin, N., Groysman, M., and Citri, A. (2017). Mapping synaptic cortico-claustral connectivity in the mouse. J. Comp. Neurol. *525*, 1381–1402.

Atlan, G., Terem, A., Peretz-Rivlin, N., Kamini, S., Gonzolas, B.J., Pozner, G.-i., Tasaka, G., Goel, Y., Refaeli, R., Zviran, O., Lim, B.K., et al. (2018). The claustrum supports resilience to distraction. Curr. Biol. Published online August 16, 2016. https://doi.org/10.1016/j.cub.2018.06.068.

Banks, M.I., White, J.A., and Pearce, R.A. (2000). Interactions between distinct GABA(A) circuits in hippocampus. Neuron *25*, 449–457.

Bruno, R.M. (2011). Synchrony in sensation. Curr. Opin. Neurobiol. *21*, 701–708.

Bruno, R.M., and Sakmann, B. (2006). Cortex is driven by weak but synchronously active thalamocortical synapses. Science *312*, 1622–1627.

Capogna, M. (2011). Neurogliaform cells and other interneurons of stratum lacunosum-moleculare gate entorhinal-hippocampal dialogue. J. Physiol. *589*, 1875–1883.

Capogna, M., and Pearce, R.A. (2011). GABA A,slow: causes and consequences. Trends Neurosci. *34*, 101–112.

Chittajallu, R., Pelkey, K.A., and McBain, C.J. (2013). Neurogliaform cells dynamically regulate somatosensory integration via synapse-specific modulation. Nat. Neurosci. *16*, 13–15.

Collins, D.P., Anastasiades, P.G., Marlin, J.J., Carter, A.G., Collins, D.P., Anastasiades, P.G., Marlin, J.J., and Carter, A.G. (2018). Reciprocal circuits linking the prefrontal cortex with dorsal and ventral thalamic nuclei article reciprocal circuits linking the prefrontal cortex with dorsal and ventral thalamic nuclei. Neuron *98*, 366–379.

Cortimiglia, R., Crescimanno, G., Salerno, M.T., and Amato, G. (1991). The role of the claustrum in the bilateral control of frontal oculomotor neurons in the cat. Exp. Brain Res. *84*, 471–477.

Courtin, J., Chaudun, F., Rozeske, R.R., Karalis, N., Gonzalez-Campo, C., Wurtz, H., Abdi, A., Baufreton, J., Bienvenu, T.C.M., and Herry, C. (2014). Prefrontal parvalbumin interneurons shape neuronal activity to drive fear expression. Nature *505*, 92–96.

Crick, F.C., and Koch, C. (2005). What is the function of the claustrum? Philos. Trans. R. Soc. B Biol. Sci. *360*, 1271–1279.

Cruikshank, S.J., Lewis, T.J., and Connors, B.W. (2007). Synaptic basis for intense thalamocortical activation of feedforward inhibitory cells in neocortex. Nat. Neurosci. *10*, 462–468.

Cruikshank, S.J., Ahmed, O.J., Stevens, T.R., Patrick, S.L., Gonzalez, A.N., Elmaleh, M., and Connors, B.W. (2012). Thalamic control of layer 1 circuits in prefrontal cortex. J. Neurosci. *32*, 17813–17823.

da Costa, N.M., Fürsinger, D., and Martin, K.A.C. (2010). The synaptic organization of the claustral projection to the cat's visual cortex. J. Neurosci. *30*, 13166–13170.

Delevich, K., Tucciarone, J., Huang, Z.J., and Li, B. (2015). The mediodorsal thalamus drives feedforward inhibition in the anterior cingulate cortex via parvalbumin interneurons. J. Neurosci. *35*, 5743–5753.

Dilgen, J., Tejeda, H.A., and O'Donnell, P. (2013). Amygdala inputs drive feedforward inhibition in the medial prefrontal cortex. J. Neurophysiol. *110*, 221–229.

Do-Monte, F.H., Quiñones-Laracuente, K., and Quirk, G.J. (2015). A temporal shift in the circuits mediating retrieval of fear memory. Nature *519*, 460–463.

Felix-Ortiz, A.C., Burgos-Robles, A., Bhagat, N.D., Leppla, C.A., and Tye, K.M. (2016). Bidirectional modulation of anxiety-related and social behaviors by amygdala projections to the medial prefrontal cortex. Neuroscience *321*, 197–209.

Floresco, S.B., and Tse, M.T. (2007). Dopaminergic regulation of inhibitory and excitatory transmission in the basolateral amygdala-prefrontal cortical pathway. J. Neurosci. *27*, 2045–2057.

Fuentealba, P., Begum, R., Capogna, M., Jinno, S., Márton, L.F., Csicsvari, J., Thomson, A., Somogyi, P., and Klausberger, T. (2008). Ivy cells: a population of nitric-oxide-producing, slow-spiking GABAergic neurons and their involvement in hippocampal network activity. Neuron *57*, 917–929.

Gabbott, P.L.A., Warner, T.A., and Busby, S.J. (2006). Amygdala input monosynaptically innervates parvalbumin immunoreactive local circuit neurons in rat medial prefrontal cortex. Neuroscience *139*, 1039–1048.

Gabernet, L., Jadhav, S.P., Feldman, D.E., Carandini, M., and Scanziani, M. (2005). Somatosensory integration controlled by dynamic thalamocortical feed-forward inhibition. Neuron *48*, 315–327.

Gerashchenko, D., Wisor, J.P., Burns, D., Reh, R.K., Shiromani, P.J., Sakurai, T., de la Iglesia, H.O., and Kilduff, T.S. (2008). Identification of a population of sleep-active cerebral cortex neurons. Proc. Natl. Acad. Sci. USA *105*, 10227–10232.

Goll, Y., Atlan, G., and Citri, A. (2015). Attention: the claustrum. Trends Neurosci. *38*, 486–495.

Gomez, J.L., Bonaventura, J., Lesniak, W., Mathews, W.B., Sysa-Shah, P., Rodriguez, L.A., Ellis, R.J., Richie, C.T., Harvey, B.K., Dannals, R.F., et al. (2017). Chemogenetics revealed: DREADD occupancy and activation via converted clozapine. Science *357*, 503–507.

Gunaydin, L.A., Grosenick, L., Finkelstein, J.C., Kauvar, I.V., Fenno, L.E., Adhikari, A., Lammel, S., Mirzabekov, J.J., Airan, R.D., Zalocusky, K.A., et al. (2014). Natural neural projection dynamics underlying social behavior. Cell *157*, 1535–1551.

Hall, S., Hunt, M., Simon, A., Cunnington, L.G., Carracedo, L.M., Schofield, I.S., Forsyth, R., Traub, R.D., and Whittington, M.A. (2015). Unbalanced peptidergic inhibition in superficial neocortex underlies spike and wave seizure activity. J. Neurosci. *35*, 9302–9314.

Hull, C., Isaacson, J.S., and Scanziani, M. (2009). Postsynaptic mechanisms govern the differential excitation of cortical neurons by thalamic inputs. J. Neurosci. *29*, 9127–9136.

Isaacson, J.S., and Scanziani, M. (2011). How inhibition shapes cortical activity. Neuron *72*, 231–243.

Jiang, X., Shen, S., Cadwell, C.R., Berens, P., Sinz, F., Ecker, A.S., Patel, S., and Tolias, A.S. (2015). Principles of connectivity among morphologically defined cell types in adult neocortex. Science *350*, aac9462.

Karagiannis, A., Gallopin, T., Dávid, C., Battaglia, D., Geoffroy, H., Rossier, J., Hillman, E.M.C., Staiger, J.F., and Cauli, B. (2009). Classification of NPY-expressing neocortical interneurons. J. Neurosci. *29*, 3642–3659.

Kawaguchi, Y., and Kubota, Y. (1997). GABAergic cell subtypes and their synaptic connections in rat frontal cortex. Cereb. Cortex *7*, 476–486.

Kim, J., Matney, C.J., Roth, R.H., and Brown, S.P. (2016). Synaptic organization of the neuronal circuits of the claustrum. J. Neurosci. *36*, 773–784.

Kitanishi, T., and Matsuo, N. (2017). Organization of the claustrum-to-entorhinal cortical connection in mice. J. Neurosci. *37*, 269–280.

Klavir, O., Prigge, M., Sarel, A., Paz, R., and Yizhar, O. (2017). Manipulating fear associations via optogenetic modulation of amygdala inputs to prefrontal cortex. Nat. Neurosci. *20*, 836–844.

Koubeissi, M.Z., Bartolomei, F., Beltagy, A., and Picard, F. (2014). Electrical stimulation of a small brain area reversibly disrupts consciousness. Epilepsy Behav. *37*, 32–35.

Krook-Magnuson, E., Luu, L., Lee, S.-H., Varga, C., and Soltesz, I. (2011). Ivy and neurogliaform interneurons are a major target of μ-opioid receptor modulation. J. Neurosci. *31*, 14861–14870.

LeVay, S. (1986). Synaptic organization of claustral and geniculate afferents to the visual cortex of the cat. J. Neurosci. *6*, 3564–3575.

LeVay, S., and Sherk, H. (1981). The visual claustrum of the cat. I. Structure and connections. J. Neurosci. *1*, 956–980.

Likhtik, E., Stujenske, J.M., Topiwala, M.A., Harris, A.Z., and Gordon, J.A. (2014). Prefrontal entrainment of amygdala activity signals safety in learned fear and innate anxiety. Nat. Neurosci. *17*, 106–113.

Lu, J., Tucciarone, J., Padilla-Coreano, N., He, M., Gordon, J.A., and Huang, Z.J. (2017). Selective inhibitory control of pyramidal neuron ensembles and cortical subnetworks by chandelier cells. Nat. Neurosci. *20*, 1377–1383.

Mathur, B.N. (2014). The claustrum in review. Front. Syst. Neurosci. *8*, 48.

McGarry, L.M., and Carter, A.G. (2016). Inhibitory gating of basolateral amygdala inputs to the prefrontal cortex. J. Neurosci. *36*, 9391–9406.

Milstein, A.D., Bloss, E.B., Apostolides, P.F., Vaidya, S.P., Dilly, G.A., Zemelman, B.V., and Magee, J.C. (2015). Inhibitory gating of input comparison in the CA1 microcircuit. Neuron *87*, 1274–1289.

Neske, G.T., Patrick, S.L., and Connors, B.W. (2015). Contributions of diverse excitatory and inhibitory neurons to recurrent network activity in cerebral cortex. J. Neurosci. *35*, 1089–1105.

Nishikawa, K., and MacIver, M.B. (2001). Agent-selective effects of volatile anesthetics on GABAA receptor-mediated synaptic inhibition in hippocampal interneurons. Anesthesiology *94*, 340–347.

Oláh, S., Füle, M., Komlósi, G., Varga, C., Báldi, R., Barzó, P., and Tamás, G. (2009). Regulation of cortical microcircuits by unitary GABA-mediated volume transmission. Nature *461*, 1278–1281.

Otis, J.M., Namboodiri, V.M.K., Matan, A.M., Voets, E.S., Mohorn, E.P., Kosyk, O., McHenry, J.A., Robinson, J.E., Resendez, S.L., Rossi, M.A., and Stuber, G.D. (2017). Prefrontal cortex output circuits guide reward seeking through divergent cue encoding. Nature *543*, 103–107.

Overstreet-Wadiche, L., and McBain, C.J. (2015). Neurogliaform cells in cortical circuits. Nat. Rev. Neurosci. *16*, 458–468.

Remedios, R., Logothetis, N.K., and Kayser, C. (2010). Unimodal responses prevail within the multisensory claustrum. J. Neurosci. *30*, 12902–12907.

Salerno, M.T., Cortimiglia, R., Crescimanno, G., Amato, G., and Infantellina, F. (1984). Effects of claustrum stimulation on spontaneous bioelectrical activity of motor cortex neurons in the cat. Exp. Neurol. *86*, 227–239.

Salerno, M.T., Cortimiglia, R., Crescimanno, G., and Amato, G. (1989). Effect of claustrum activation on the spontaneous unitary activity of frontal eye field neurons in the cat. Neurosci. Lett. *98*, 299–304.

Saunders, A., Oldenburg, I.A., Berezovskii, V.K., Johnson, C.A., Kingery, N.D., Elliott, H.L., Xie, T., Gerfen, C.R., and Sabatini, B.L. (2015). A direct GABAergic output from the basal ganglia to frontal cortex. Nature *521*, 85–89.

Seiriki, K., Kasai, A., Hashimoto, T., Schulze, W., Niu, M., Yamaguchi, S., Nakazawa, T., Inoue, K.I., Uezono, S., Takada, M., et al. (2017). High-speed and scalable whole-brain imaging in rodents and primates. Neuron *94*, 1085–1100.

Simon, A., Oláh, S., Molnár, G., Szabadics, J., and Tamás, G. (2005). Gap-junctional coupling between neurogliaform cells and various interneuron types in the neocortex. J. Neurosci. *25*, 6278–6285.

Smith, J.B., Radhakrishnan, H., and Alloway, K.D. (2012). Rat claustrum coordinates but does not integrate somatosensory and motor cortical information. J. Neurosci. *32*, 8583–8588.

Stachniak, T.J., Ghosh, A., and Sternson, S.M. (2014). Chemogenetic synaptic silencing of neural circuits localizes a hypothalamus→midbrain pathway for feeding behavior. Neuron *82*, 797–808.

Sternson, S.M., and Roth, B.L. (2014). Chemogenetic tools to interrogate brain functions. Annu. Rev. Neurosci. *37*, 387–407.

Szabadics, J., Tamás, G., and Soltesz, I. (2007). Different transmitter transients underlie presynaptic cell type specificity of GABAA,slow and GABAA,fast. Proc. Natl. Acad. Sci. USA *104*, 14831–14836.

Tamás, G., Lörincz, A., Simon, A., and Szabadics, J. (2003). Identified sources and targets of slow inhibition in the neocortex. Science *299*, 1902–1905.

Tervo, D.G.R., Hwang, B.Y., Viswanathan, S., Gaj, T., Lavzin, M., Ritola, K.D., Lindo, S., Michael, S., Kuleshova, E., Ojala, D., et al. (2016). A designer AAV variant permits efficient retrograde access to projection neurons. Neuron *92*, 372–382.

Tovote, P., Fadok, J.P., and Lüthi, A. (2015). Neuronal circuits for fear and anxiety. Nat. Rev. Neurosci. *16*, 317–331.

Tricoire, L., Pelkey, K.A., Daw, M.I., Sousa, V.H., Miyoshi, G., Jeffries, B., Cauli, B., Fishell, G., and McBain, C.J. (2010). Common origins of hippocampal Ivy and nitric oxide synthase expressing neurogliaform cells. J. Neurosci. *30*, 2165–2176.

van den Pol, A.N., Yao, Y., Fu, L.-Y., Foo, K., Huang, H., Coppari, R., Lowell, B.B., and Broberger, C. (2009). Neuromedin B and gastrin-releasing peptide excite arcuate nucleus neuropeptide Y neurons in a novel transgenic mouse expressing strong Renilla green fluorescent protein in NPY neurons. J. Neurosci. *29*, 4622–4639.

Wang, Q., Ng, L., Harris, J.A., Feng, D., Li, Y., Royall, J.J., Oh, S.W., Bernard, A., Sunkin, S.M., Koch, C., and Zeng, H. (2017). Organization of the connections between claustrum and cortex in the mouse. J. Comp. Neurol. *525*, 1317–1346.

White, M.G., Cody, P.A., Bubser, M., Wang, H.D., Deutch, A.Y., and Mathur, B.N. (2017). Cortical hierarchy governs rat claustrocortical circuit organization. J. Comp. Neurol. *525*, 1347–1362.

Xu, X., Roby, K.D., and Callaway, E.M. (2010). Immunochemical characterization of inhibitory mouse cortical neurons: three chemically distinct classes of inhibitory cells. J. Comp. Neurol. *518*, 389–404.

Yizhar, O., Fenno, L.E., Prigge, M., Schneider, F., Davidson, T.J., O'Shea, D.J., Sohal, V.S., Goshen, I., Finkelstein, J., Paz, J.T., et al. (2011). Neocortical excitation/inhibition balance in information processing and social dysfunction. Nature *477*, 171–178.

Zingg, B., Hintiryan, H., Gou, L., Song, M.Y., Bay, M., Bienkowski, M.S., Foster, N.N., Yamashita, S., Bowman, I., Toga, A.W., and Dong, H.W. (2014). Neural networks of the mouse neocortex. Cell *156*, 1096–1111.

STAR★METHODS

KEY RESOURCES TABLE

REAGENT or RESOURCE	SOURCE	IDENTIFIER
Antibodies		
Goat anti-parvalbumin	Swant	Cat# Pvg213; RRID: AB_10000345
Rabbit anti-NPY	Abcam	Cat# 30914; RRID: AB_1566510
Chicken Anti-GFP	Aves Labs	Cat# GFP-1020; RRID: AB_10000240
Donkey anti-goat Alexa Fluor-647	Thermoscientific	Cat# A-21447; RRID: AB_2535864
Donkey anti-rabbit Alexa Fluor-594.	Thermoscientific	Cat# A-21207; RRID: AB_2556547
Bacterial and Virus Strains		
AAVretro-CAG-tdTomato	Janelia Virus services	N/A
AAVretro-Syn-cre	Janelia Virus services; Tervo et al., 2016	N/A
AAV-EF1a-DIO-hChR2(H134R)-EYFP	UNC vector core	N/A
AAV5-CAG-DIO-hM4d-gfp	Janelia Virus services	N/A
AAV1.hSyn.ChR2(H134R)-eYFP.WPRE.hGH	UPENN	N/A
Chemicals, Peptides, and Recombinant Proteins		
CNQX	Sigma Aldrich	Cat# C127
APV	Caymen chemical	Cat#14539
Gabazine (SR 95531)	Sigma Aldrich	Cat# S106
CNO	Enzo life sciences	Cat# BML-NS105-0005
Vectashiled	Vectorlabs	Cat# H-1000
Prolong gold	Thermofisher	Cat# P36931
Experimental Models: Organisms/Strains		
C57BL/6N	Charles River, https://www.criver.com/	N/A
NPY-ires-Cre	Janelia Gene Targeting and Transgenics	N/A
NPYhr-GFP	Jackson Laboratory	RRID: I MSR_JAX:006417
PV-ires-cre	Jackson Laboratory	RRID: IMSR_JAX:008069
SOM-ires-cre	Jackson Laboratory	RRID: IMSR_JAX:013044
VIP-cre	Jackson Laboratory	RRID: IMSR_JAX:010908
Ai9	Jackson Laboratory	RRID: IMSR_JAX:007909
Rat/Long Evans	Charles River, https://www.criver.com/	N/A
Software and Algorithms		
ImageJ	NIH	https://imagej.nih.gov/ij/
MATLAB	Mathworks	N/A
Other		
Fiber optic cannulas	Doric Lens	MFC_200/245-0.37_4mm_ZF1.25
Microelectrodes	Microprobes	Cat# WE30030.5A3

CONTACT FOR REAGENT AND RESOURCE SHARING

Further information and requests for resources and reagents should be directed to and will be fulfilled by the Lead Contact, Albert K. Lee (leea@janelia.hhmi.org).

EXPERIMENTAL MODELS AND SUBJECT DETAILS

All procedures were conducted in accordance with protocols approved by the Janelia Institutional Animal Care and Use Committee and The Crick Institute animal welfare committee. Mice (60-200 days old) of both sexes were used for *in vivo* and *in vitro* electrophysiology and neuronal tracing. Male Long-Evans rats weighing 300-400 g were also used. Rats and mice were housed in a temperature

controlled environment on a reverse 12-12 hour light-dark cycle. Rats and C57BL6 mice were obtained from Charles River Laboratories. PV-Cre, SOM-Cre, VIP-Cre, NPY-Cre, and VGAT-cre, NPYhr-GFP, and Ai9 reporter mice were obtained from Jackson Laboratory (https://www.jax.org/) and bred at Janelia Research Campus, and NPY-Cre (Milstein et al., 2015) were generated and bred at the Janelia Research Campus. Ai9 mice were crossed with PV-cre, SOM-cre, NPY-cre and VGAT-cre mice to generate PV-cre-TOM, SOM-cre-TOM, NPY-cre-TOM, and VGAT-cre-TOM mice where the respective interneuron populations were labeled with tdTomato.

METHOD DETAILS

Viruses and surgery

Mice were injected with 150-200 nl of AAVretro-syn-Cre or AAVretro-CAG-tdTomato (Tervo et al., 2016) into the PFC, specifically targeting areas of the anterior cingulate and prelimbic cortex. Anterior-posterior (A/P) and medio-lateral (M/L) coordinates are measured relative to bregma, and all dorsal-ventral (D/V) coordinates are from brain surface at the site of injection. PFC coordinates were A/P: 1.7 mm, M/L: 0.4 mm, and D/V: −1.5 mm and −0.5 mm. The PFC injections in rats were made with a single injection at A/P: 3.0 mm, M/L: 1.0 mm, D/V: 2.0 mm. The CLA injections in mice were made with a single injection at A/P: 1.3 mm, M/L: 2.4 mm, and D/V: −2.3-2.5 mm. The CLA injections in rats were made at A/P: 1.5 mm, M/L, 4.5 mm, and D/V: 5.0 mm. Basolateral amygdala injections in mice were made bilaterally at A/P: −1.5 mm, M/L 3.0 mm, and D/V: −3.0 mm. For medio-dorsal (MD) thalamus experiments in mice, virus was injected at A/P:-1.6 mm, M/L: 0.5 mm, D/V: −3.0 mm. The MD injections in rats were made at A/P: −2.5 mm, M/L: 1.0 mm, D/V: −5.5 mm. AAV5-EF1a-DIO-hChR2-EYFP (UNC) (100-120 nl) or AAV1-syn-ChR2-eYFP (30-40 nl) was injected into the CLA. For the direct activation of PV or NPY cells *in vivo*, AAV5-EF1-DIO-hChR2-EYFP (300 nl) was injected into the PFC of either PV-Cre or NPY-Cre mice. For slice physiology experiments, AAV1-syn-ChR2(H134R)-eYFP (UPENN Vector core) was injected in small volumes (30-40 nl) into the CLA. In some of these experiments we observed some virus leakage into the neighboring ventrolateral striatum. However, as the neighboring striatum does not project to the PFC, and our cannula was located over the CLA, we included these animals. If no CLA fibers were observed in the PFC, or if the injection was predominantly in the striatum, the animal was removed from the study. For interneuron suppression experiments, 300 nl of AAV5-CAG-FLEX-hM4D-GFP (Janelia Research Campus Virus Services) was injected into the PFC coordinates of mice at two depths (−1.5 mm and −0.5 mm from brain surface) in PV-Cre, SOM-Cre, VIP-Cre, VGAT-Cre, and NPY-Cre mice, together with 30-40 nl of AAV1-syn-ChR2-eYFP into the CLA. Experiments were performed after 4-6 weeks of recovery time. For all mice, 5 mg/kg of CNO (Enzo Life Sciences) was administered to activate the hM4D receptor.

In vivo physiology

For awake recordings, animals with previous virus injections were implanted chronically with optic fibers and a head plate for head fixation, and singly housed with a running wheel. Habituation of head fixation was initiated 7-10 days following surgery. Animals were head-fixed and allowed to run on a custom-made linear treadmill. Animals were habituated to the treadmill for twenty minutes to one hour each day for 3-5 days. After habituation, a small craniotomy was made over the PFC region where the initial AAVretro-syn-Cre injection had been made (1.7 mm anterior to bregma). Sharpened tungsten microelectrodes (tapered 2 μm tip, ~0.5 MΩ, Microprobes, Gaithersburg, Maryland) were advanced into the PFC using stereotaxic coordinates. The electrode was advanced and single neuron or multi-neuron recordings were made at several depths. At this medio-lateral location, mainly layer 2-3 neurons were sampled from anterior cingulate, and layer 5 neurons were sampled from prelimbic cortex. The dependence of neural responses as a function of depth along this axis is reported in Figure S1. Several days (3-8 days) of recording could be obtained from the same animal. Signals were referenced to a screw placed in the cerebellum and filtered between 1 Hz – 10 kHz or 0.3 kHz – 10 kHz (A-M systems, model 1700), then sampled at 25 kHz (Heka ITC-1600 and Patchmaster). For interneuron suppression experiments using AAV5-FLEX-hM4D-GFP, animals were injected with virus (as described above), and after 4-6 weeks, mice were lightly anesthetized with isoflurane (0.9%–1% isoflurane and 1.5% oxygen) and an optic fiber inserted acutely into the left CLA while recording responses in the left PFC. After obtaining recordings to confirm CLA evoked inhibition, CNO was delivered (5 mg / kg, I.P. in saline), and the resulting CLA-mediated responses were obtained. Recordings were only used when they were obtained less than 1.5 hours following CNO delivery. No effect of CNO was observed in VIP-Cre-hM4D and SOM-Cre-hM4D experiments, so these experiments also served as CNO controls, confirming that CNO did not have a direct influence on CLA evoked inhibition.

Optogenetic activation

Light stimulation was performed using a 473 nm laser (Shanghai Dream Laser), using TTL pulses controlled by a Master-9 (AMPI). For the 0.2 mm, 0.37 NA fibers (Doric Lenses, Quebec City, Canada) used *in vivo*, light measured from the fiber tip was < 10mW, and in most cases 7 mW. Five-millisecond pulses were used for CLA activation *in vivo* and delivered at a frequency of 0.2 Hz. Higher frequencies of stimuli (1 Hz – 20 Hz) were also used for some recordings after the initial 0.2 Hz frequency stimuli were delivered. For experiments testing the direct activation of PV and NPY interneurons, light (1-5 ms, 2-3 mW) was delivered through a 0.1 mm optic fiber attached to the recording electrode ~0.5 mm above the recording tip. Light stimuli were synchronized with the recordings and stored for analysis offline. For *in vitro* slice recordings, light was delivered through the microscope objective (Olympus 40x 0.8 NA water immersion) through a GFP excitation filter cube and pulses were controlled by a fluorescence unit (Sutter Lamdba DG-4). Light intensity was 1.8 mW at the specimen. For current clamp experiments, 3 ms pulses were used, and for voltage clamp recordings

50-100 ms pulses were used. Only the initial amplitude and latency (< 20 ms) response was analyzed in the case of voltage clamp experiments.

In vitro slice preparation and recording

Coronal brain slices from P67-120 animals were prepared after cervical dislocation and decapitation. The brain was rapidly dissected and cooled in continuously gassed (95% O_2 and 5% CO_2), icy cutting solution containing (in mM): 90 N-methyl-D-glucamine, 20 HEPES, 110 HCl, 3 KCl, 10 $MgCl_2$, 0.5 $CaCl_2$, 1.1 NaH_2PO_4, 25 $NaHCO_3$, 3 pyruvic acid, 10 ascorbic acid and 25 D-glucose. 350 μm thick coronal brain slices were cut on a vibratome (Microm) and allowed to recover for 15 min at 37°C in cutting solution, then transferred to 22°C in standard artificial cerebrospinal fluid (ACSF) containing (in mM): 126 NaCl, 3 KCl, 2 $MgSO_4$, 2 $CaCl_2$, 1.1 NaH2PO_4, 26 $NaHCO_3$, 0.1 pyruvic acid, 0.5 L-glutamine, 0.4 ascorbic acid and 25 D-glucose, continuously gassed with 95% O_2 and 5% CO_2. Patch-clamp recordings were performed in a submerged chamber with 5-10 ml/min superfusion of ACSF. 4-7 MΩ patch pipettes were filled with intracellular solution containing (in mM): 130 K-gluconate, 5 NaCl, 2 $MgSO_2$, 10 HEPES, 0.1 EGTA, 4 Mg-ATP, 0.4 Na-GTP, 7 phosphocreatine, 2 pyruvic acid, 0.1 Alexa594, 0.2% biocytin, and ~10 mM KOH (to set pH to 7.3). Gabazine (SR-95531, Sigma), CNQX (Sigma) and D-AP5 (Caymen Chemical) were dissolved in ACSF. Whole-cell recordings were not analyzed if the access resistance was > 25 MΩ. Cells were identified with an upright microscope with 40X water immersion objective (0.8 NA, Olympus) and fluorescence optics. Voltage and current recordings were low-pass filtered at 3.3 kHz and sampled at 10 kHz (HEKA EPC10 amplifier and Patchmaster). Recordings were performed at 32°C.

Immunohistochemistry

Mice were perfused through the heart with phosphate buffered saline (PBS), followed by 4% paraformaldehyde (PFA). Brains were stored in PFA for 24-48 hours before sectioning (50 μm) on a vibrotome. Slices were washed three times with PBS and incubated in blocking buffer (2% bovine serum + 0.4% triton) for one hour, then incubated in primary antibodies + blocking buffer for 18-24 hours at room temperature. Secondary antibodies were applied for 3-4 hours in the blocking buffer and slices were washed, mounted, and coverslipped using Vectashield with DAPI or ProLong Gold (Life Technologies). Images were collected on a TissueGnostics slide scanner using a 10x objective with wide field illumination, or a 20x objective for confocal images, and analyzed in ImageJ and MATLAB using built-in toolboxes. Primary antibodies included chicken anti-GFP (1:1000, Aves Labs), and goat anti-PV (1:1000, Swant), and rabbit anti-NPY (1:500, Abcam Catalog #30194). Secondary antibodies (Thermo Fisher Scientific) were used at a concentration of 1:500, and included donkey anti-chicken Alexa Fluor-488, donkey anti-goat Alexa Fluor-647, and donkey anti-rabbit Alexa Fluor-594.

Analysis of axon density

Following expression of DIO-ChR2 for 4-8 weeks, animals were perfused with ice cold PBS, and 4% paraformaldehyde. Brain tissue was cut in 50 μm coronal sections, washed with PBS, incubated in 0.4% Triton-X and bovine serum (BSA, 2%) for 1h followed by 24-hour incubation in chicken anti – GFP (Aves Labs) at 4°C. Anti-chicken Alexa Fluor-488 was used as the secondary antibody and slices were incubated for 24 hours at 4°C. The tissue was then mounted with Vectashield or ProLong Gold, stained with DAPI, and imaged on a TissueGnostics slide scanner. Images were analyzed in ImageJ, and the ChR2 axon density was measured as the mean fluorescence across a 200 μm line profile drawn from the white matter to pia, or through the BLA, hippocampus, or striatum. The axon density was calculated as a function of white matter to PIA distance.

Analysis of electrophysiology data

All data were analyzed in MATLAB using custom scripts and functions. For *in vivo* spiking, data were band – pass filtered between 0.3 kHz-5 kHz, and events with a signal to noise ratio of at least 4 were detected and treated as multiunit activity or single unit activity depending on the interspike interval distribution. Units with > 0.1% of events occurring with an interspike interval < 2ms were considered multiunit, and others were considered single unit. Spike widths were calculated as the time from spike peak to trough, and putative interneurons were considered to be those with < 0.4 ms widths. The CLA modulation index (MI) was calculated as $(FR_{post} - FR_{pre}) / (FR_{post} + FR_{pre})$, thus a value of −1 would be complete suppression, and a value approaching 1 strong activation. The FR_{pre} was taken as the mean firing rate in the 1 s prior to CLA activation, and FR_{post} as the mean firing rate from 0-0.1 s after CLA activation. Note that this metric can still yield negative values even if the cell is initially excited. Therefore, we also report the percentage of recordings showing excitation. Spike recordings were considered to show excitation if any 5 ms bin in the 30 ms period following CLA activation had a firing rate greater than 4 standard deviations above the mean baseline firing rate. To determine the latency of spike activation for interneurons, 1 ms bins were used, and the peak in the PSTH following CLA activation was detected. For temporal jitter analysis and spike probability, the first spike in the 2-20 ms period following CLA activation was used. For presentation, data were normalized by dividing all bin values of the PSTH by the mean firing rate in the 2 s prior to CLA stimulation. In Figure 1H, the cells were sorted according to their firing rate in the 200-500 ms period post CLA activation, and the upper and lower quartiles were used to identify late activated and late suppressed cells.

For *in vitro* patch-clamp recording analysis, trials where single optogenetic pulses (3 ms, 0.2 Hz), or 20 Hz pulses (3 ms pulses, delivered every 10 s) were analyzed. The mean voltage- or current-clamp response was averaged over 5-50 trials. For determining the latency to optical activation, voltage-clamp responses were obtained while cells were held at −60 mV. Only cells with at a mean

response (10-50 trials of 100 ms light activation) of > 10 pA were used to calculate latency. The onset latency was calculated as the start of the 10%–90% rise in the mean response. Since in SOM-Cre mice some FS-PV cells are also labeled, SOM-TOM cells and VGAT-TOM cells with high frequency firing (> 100 Hz with supra-threshold current injection) and narrow spike widths (< 0.4 ms) were treated as fast spiking (FS) neurons. NPY cells had a mean spike width of 1.2 ± 0.03 ms and never had a spike width < 0.6 ms.

For obtaining connectivity measurements, 50 Hz bursts of spiking were driven in the presynaptic cell, while measuring the change in voltage in 1-3 other neurons. The 50 Hz burst was used to ensure the detection of small facilitating connections and avoid false negatives. For the analysis of kinetics and magnitudes of IPSPs, a subset of cell pairs was tested using only single action potentials in the presynaptic cell. The analysis of NPY cell firing characteristics were performed on 'just above spike threshold' and 'suprathreshold' depolarization current steps. The adaptation ratio for EPSPs during 20 Hz CLA axon stimulation was calculated as PSP_{last}/PSP_{first}, where the PSPs refer to the postsynaptic potential (in mV) of the first and last pulse.

QUANTIFICATION AND STATISTICAL ANALYSIS

Wilcoxon rank sum tests or paired t tests were used for unpaired and paired data (respectively). All tests were considered significant if the p value was < 0.05. For multiple comparisons, the threshold for statistical significance was adjusted using the Bonferroni-Holm correction; however, the original p value is reported throughout.

DATA AND SOFTWARE AVAILABLILTY

Data presented in the manuscript are available from the corresponding authors upon request.

Neuron, Volume *99*

Supplemental Information

Inhibitory Control of Prefrontal Cortex by the Claustrum

Jesse Jackson, Mahesh M. Karnani, Boris V. Zemelman, Denis Burdakov, and Albert K. Lee

Figure S1: Claustrum axons throughout the brain and PFC pyramidal cell and interneuron responses to CLA activation (related to Figure 1).

A: Brain atlas images taken from The Mouse Brain in Stereotaxic Coordinates, 3rd ed., Franklin and Paxinos (Academic Press, 2007). Black boxes indicate the regions that are shown below, with CLA axons containing ChR2, and stained for GFP. ACC = anterior cingulate; M2 =secondary motor cortex; RSC = retrosplenial cortex; BLA = basolateral amygdala; S1 = primary somatosensory cortex; A1 = primary auditory cortex; V1 = primary visual cortex; POS = postsubiculum; PRC = perirhinal cortex; LEC = lateral entorhinal cortex.

B: The distribution of PFC projecting CLA collaterals in 18 different brain regions, averaged across all layers. Control brain regions (striatum, hippocampus and piriform cortex) are shown for reference, as these regions do not receive CLA inputs. Axon intensity in all regions are normalized to the mean GFP intensity in the control regions.

C: Single trial traces of three different interneurons are shown aligned to optogenetic activation of the CLA (blue) in awake mice. These cells are excited by stimulation, and then subsequently suppressed. Note these activated cells begin firing as early as 6ms following CLA activation, and in some cases, cells can fire doublets or triplets of spikes at ~200 Hz.

D: Late activated PCs had a significantly lower baseline firing rate (2.9±0.6 Hz) than late suppressed PCs (5.2±0.9 Hz, $z = 2.3$, $p = 0.02$).

E: There was no significant correlation between the baseline firing rate and the CLA modulation index for PCs.

F: The baseline firing rate was not significantly different between interneurons that were excited and interneurons that were inhibited by CLA activation

G: There was no significant correlation between the baseline firing rate and the CLA modulation index for INs.

H: The distance from the cortical surface was not significantly different between late activated and late suppressed PCs.

I: There was no significant correlation between the PC recording depth and the CLA modulation index.

J: A trend was observed in which the interneurons excited by CLA activation were located in deeper cortical layers relative to inhibited interneurons.

K: A significant correlation between CLA modulation and recording depth was detected for interneurons.

Figure S2: Optogenetic activation of the BLA and MD (related to Figure 1)

A: Schematic shows the injection of AAVretro-syn-Cre into the PFC + AAV5-EF1a-DIO-ChR2-eYFP into the BLA. On the right, the histological coronal sections show the PFC and the injection site in the BLA.

B: The mean PSTH showing the response of PFC PC cells to optogenetic stimulation of the BLA.

C: Schematic showing the injection of AAV1-syn-ChR2 into the dorsomedial thalamus (MD) of a rat. On the right, the histological sections show the PFC and the injection site in the MD.

D: The mean PSTH showing the response of PFC PC cells to optogenetic stimulation of the MD. Data are pooled across recordings from rats and mice.

E: The mean PSTH (all PCs and INs) for control experiments in mice in which AAVretro-syn-Cre was injected to the PFC and AAV5-DIO-eYFP was injected into the CLA.

Laser pulse timing and duration are indicated by the blue line in each panel. The modulation index values of the individual recordings that constitute the average responses in B, C, and E are shown as points in Figure 1L.

Figure S3: Examples of NPY-NGF neurons and NPY cells that exhibited bursting (related to Figure 2 and Figure 3)

A: Example NPY cells showing the response to hyperpolarization (black), just past spike threshold depolarization (green), and >2x spike threshold depolarization (gray). Examples shown in i – iii were not considered NPY-NGF cells as they had rapidly adapting spike trains, fired spikes early during 'just past threshold depolarizations', a hyperpolarization sag, and often showed rebound spiking following hyperpolarization. Cells in iv – viii were considered NPY-NGF cells as they had regular spiking properties and often showed later spiking in response to 'just past threshold depolarization'.

B: The separation between cells classified as putative NPY-NGF and NPY-burster is shown. Cells with an adaptation index of > -0.2 were considered as candidates for NPY-NGF cells. The adaptation index was defined using the strongest depolarization current step and computed as (FR_{late} – FR_{early}) / (FR_{late}+ FR_{early}). The more negative values have greater firing rate adaptation. FR_{early} is the firing rate in the first 500ms and FR_{late} is the firing rate in the last 500ms of the current injection.

C-D: The comparison of EPSC magnitude (C) EPSP magnitude (D) in response to CLA optogenetic activation for each NPY cell type.

E: The latency of each NPY cell type to optical CLA activation as measured in voltage-clamp. The NPY-NGF cells are active earlier.

F: The probability that each cell type will exhibit action potentials in response to CLA activation. Only 1 / 9 NPY-burster cells spiked whereas 5/17 NPY-NGF cells showed spikes. Of the NPY-NGF cells which showed definitive 'late spiking', 4/11 showed spiking in response to CLA activation.

G: The EPSP amplitude for pyramidal cells recorded simultaneously with different interneuron sub-types. No significant difference was found, suggesting the different excitatory responses of interneuron sub-types was not due to overall differences in excitability between experiments.

H: The EPSP amplitude as a function of distance from Pia. PV cells (magenta), NPY cells (green), and all neurons recorded (black) are shown. The Pia measurement was the shortest path to the pia mater from the recording electrode in vitro, whereas *in vivo* (Figure 1, Figure S1) the Pia distance was from the brain surface.

Figure S4: Mean responses of PFC pyramidal cells and FS interneurons to CLA activation before and after pharmacogenetic suppression of different interneuron subtypes (related to Figure 4).

A-E: The mean PC spiking PSTHs during CLA activation pre and post suppression of different interneuron subtypes. Data are presented as the median ± interquartile range. The expanded view in C for the PV-hM4D case shows the weak and brief increased excitation following PV cell suppression. NPY inhibition likely arrives shortly after to rapidly suppress any further excitation. Only the median response is plotted in this panel for clarity.

F: PSTH of FS interneurons *in vivo* during optogenetic CLA activation. Note that only a brief increase in spiking is observed.

G: The PSTH of pyramidal cells during optogenetic CLA activation (as shown in Figure 4E).

H: The PSTH of pyramidal cells during brief (1-5ms) optogenetic PV activation with ChR2.

I: The overlaid PSTH data from G and H. Note that the suppression lasts much shorter with PV activation than with CLA activation.

J: PSTH of FS interneurons *in vivo* during optogenetic CLA activation, following the suppression of NPY interneurons using AAV5-FLEX-hM4d + CNO. Note that PV firing is increased for a much longer duration than in F, where NPY neurons are intact.

K: The PSTH of pyramidal cells during optogenetic CLA activation, following the suppression of NPY interneurons using AAV5-FLEX-hM4d + CNO (as shown in Figure 4E).

L: The PSTH of pyramidal cells during long lasting (80ms) optogenetic PV activation with ChR2. The pulse train used was a 50 Hz train for 80ms. This long lasting PV activation is meant to approximate the extended duration of increased PV activity in J, following the suppression of NPY interneurons.

M: The overlayed PSTH data from K and L. Note the extended duration of inhibition with long lasting PV activation that matches the inhibition observed in pyramidal cells after their initial excitation in the NPY suppression experiments. The median and 25th and 75th percentile of the data are plotted in the PSTHs.

N: The proposed model of how pyramidal cell inhibition is generated by NPY cells by feedforward inhibition.

O: The proposed model of how pyramidal cell inhibition is generated by feedback inhibition from FS-PV cells, following the suppression of NPY cells.

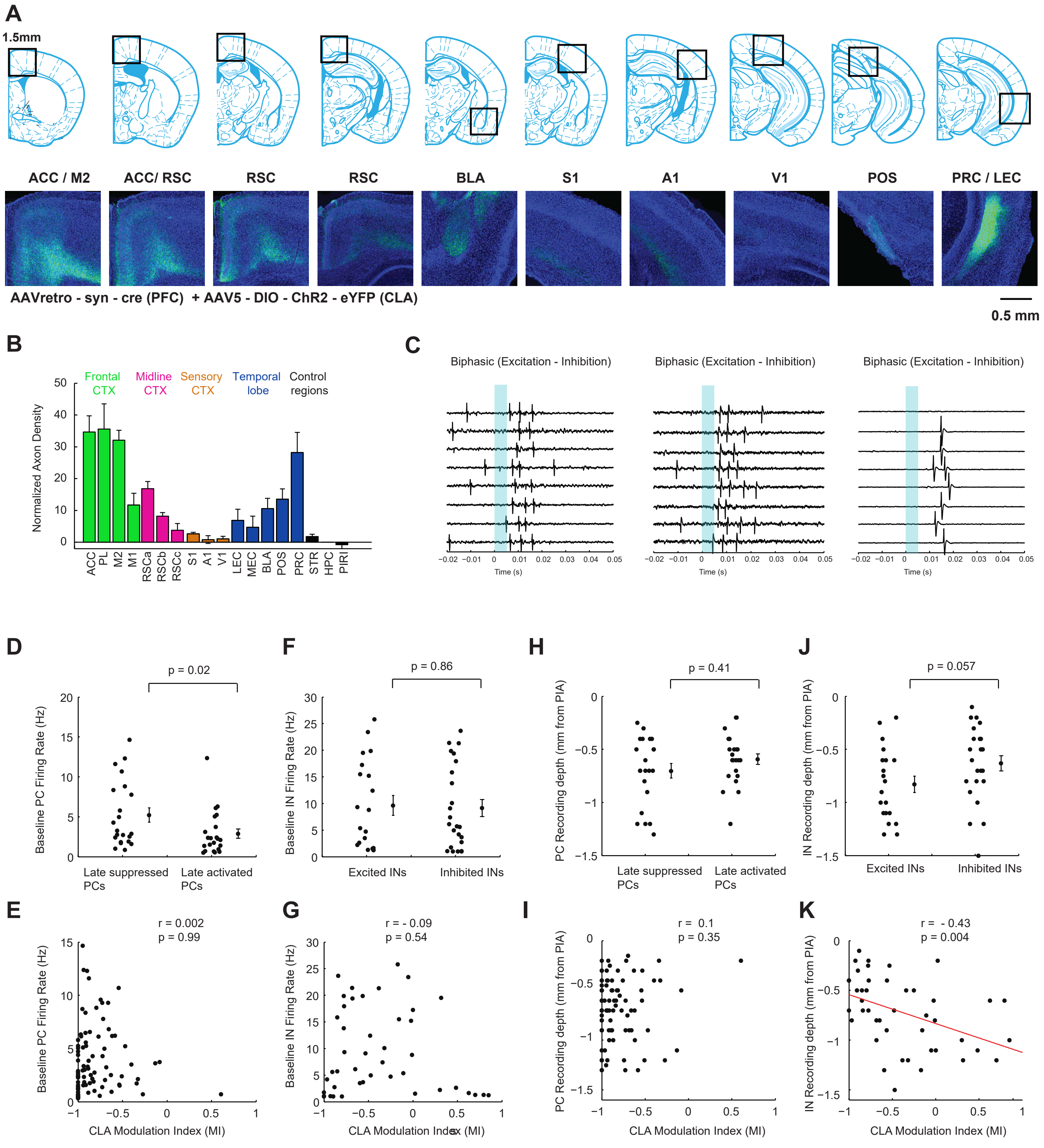

Figure S1. Related to Figure 1.

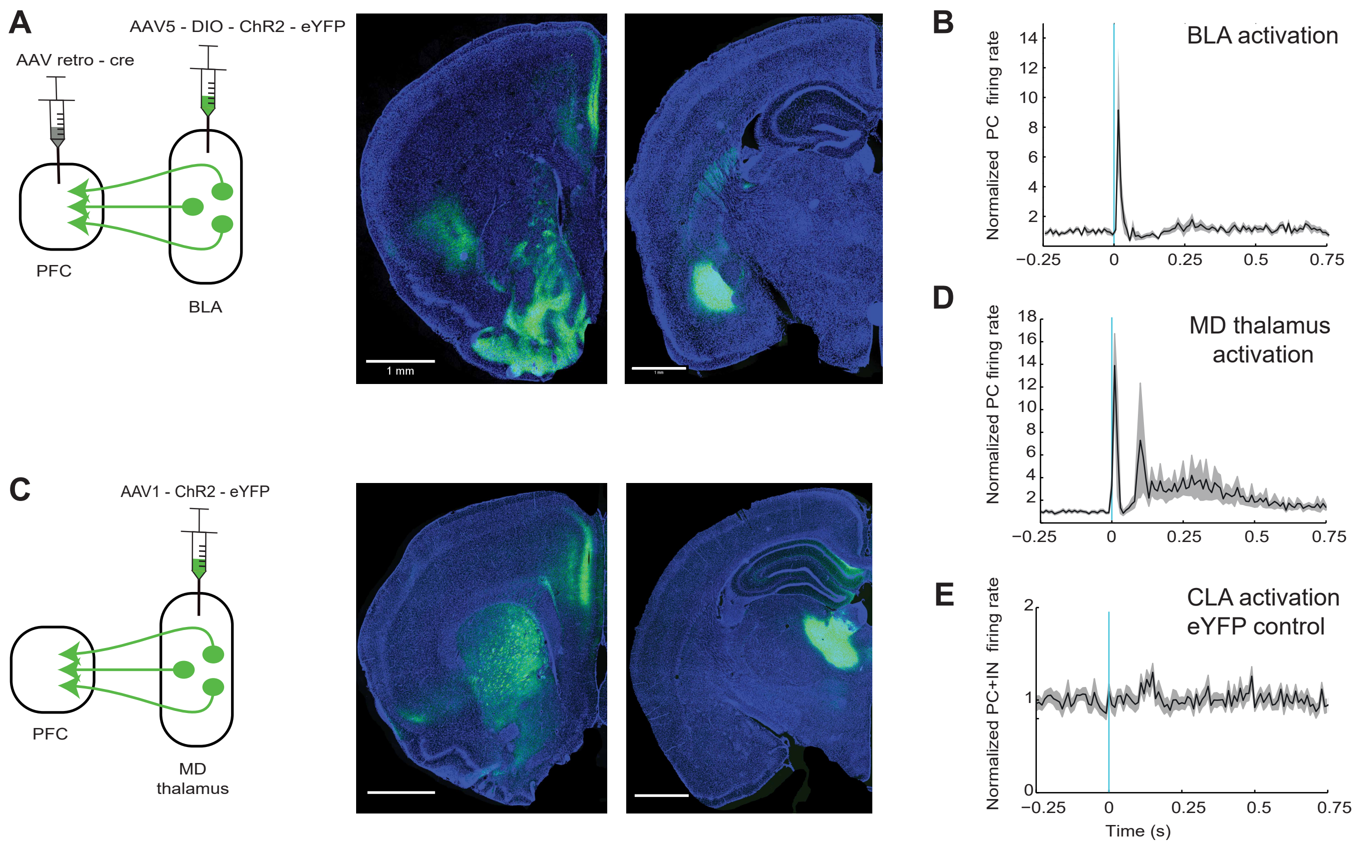

Figure S2. Related to Figure 1.

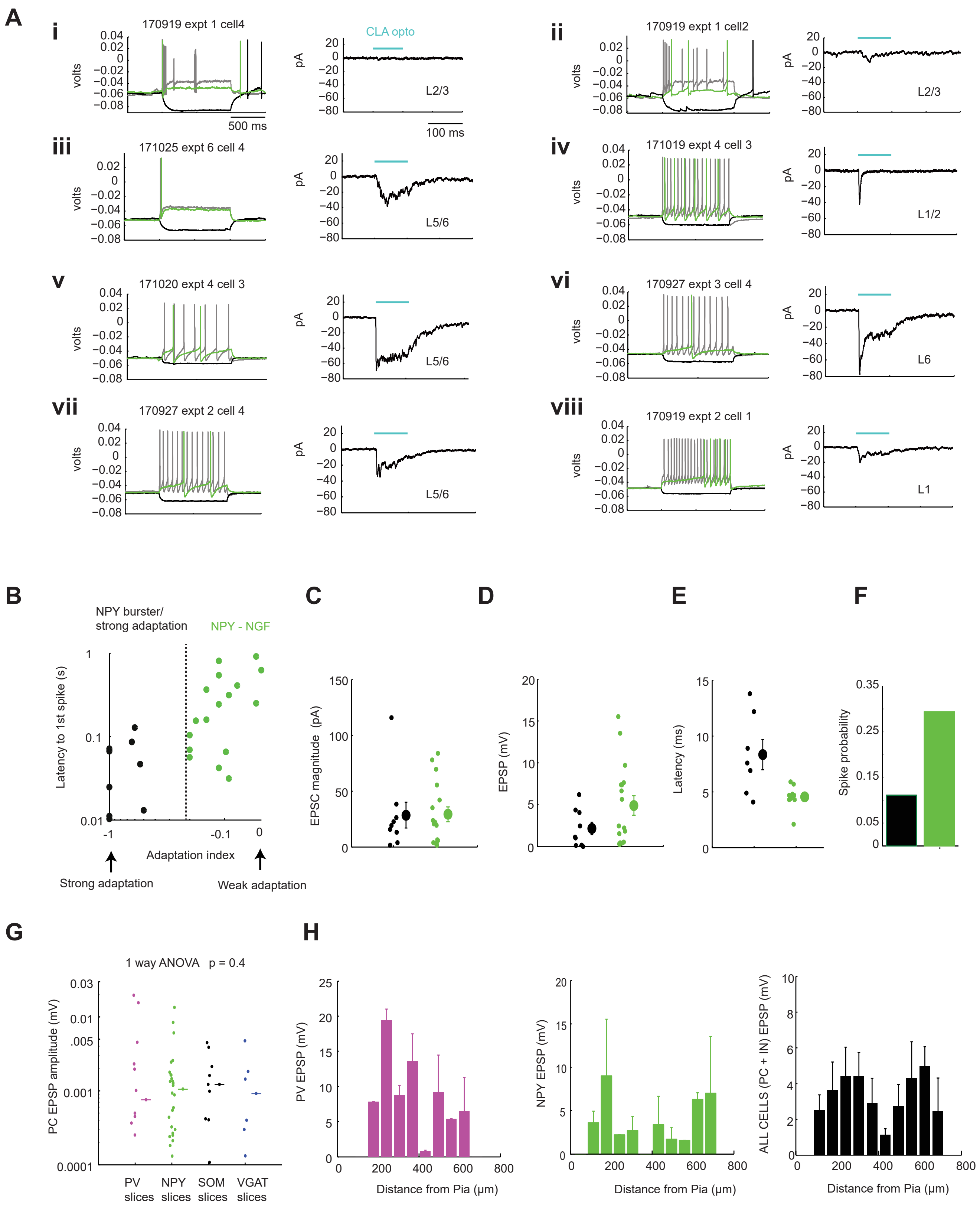

Figure S3. Related to Figure 2 and 3.

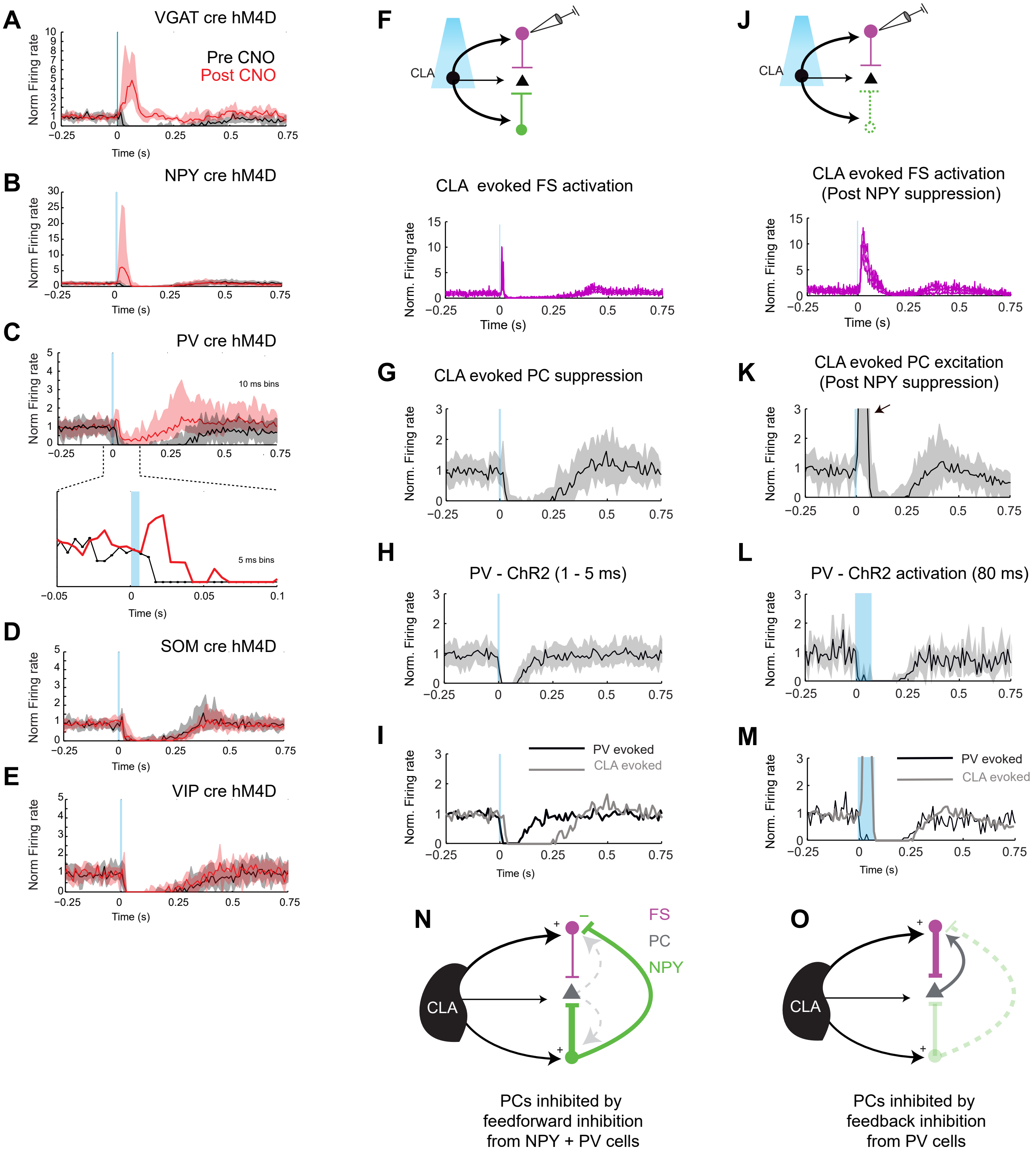

Figure S4. Related to Figure 4.
